# Does Metal Matter: Comparing Photophysical Properties of Bis-Cyclometalated Alkynylphosphonium Au(III) and Pt(II) Complexes

**DOI:** 10.3390/molecules30112434

**Published:** 2025-06-02

**Authors:** Maksim Luginin, Aleksandra Paderina, Anastasia Sizova, Elena Tupikina, Elena Grachova

**Affiliations:** Institute of Chemistry, St. Petersburg University, Universitetskii pr. 26, 198504 St. Petersburg, Russia; m.luginin@spbu.ru (M.L.); a.paderina@spbu.ru (A.P.); a.a.sizova@spbu.ru (A.S.); e.tupikina@spbu.ru (E.T.)

**Keywords:** platinum complex, gold complex, alkynylphosphonium ligand, luminescence, DFT calculations, Pt–H hydrogen bond

## Abstract

In this work, two series of Au(III) and Pt(II) alkynylphosphonium complexes of composition [M(CNC)(C_2_−L−P(CH_3_)Ph_2_)]^n+^ **Pt1**–**Pt3** (n = 0) and **Au1**–**Au3** (n = 1), (CNC = 2,6-diphenylpyridine; L = phenyl, biphenyl, naphthyl) were synthesized and characterized to discover the similarities and differences in photophysical properties between isoelectronic metallocentres. It is shown that Au(III) and Pt(II) complexes obtained demonstrate different photophysical properties despite isoelectronic metal centres, and some reasons for that are discussed based on experimental data and quantum-chemical calculation results. Complex **Pt1** also demonstrated the first example of room-temperature solution phosphorescence in the family of [Pt(CNC)(alkynyl)] complexes. It has been found that the crystal packing of **Pt1** contains a Pt–H interaction, qualified by quantum-chemical calculations as a unique hydrogen bond.

## 1. Introduction

Pt(II) complexes are widely studied because of their rich photophysical properties. They can find applications in various fields, among them OLED devices [1], photocatalysis [2], sensors [3], bioimaging [4] and even anti-counterfeiting [5]. Over the past few years, a growing interest in their isoelectronic analogues, Au(III) complexes, is emerging [6,7]. Although Au(III) complexes have long been considered non-luminescent due to low-lying d-d states [8], cyclometalated species do demonstrate interesting photophysical properties, e.g., TADF and TSDP [9,10,11] and can also be used in OLED devices [11,12].

**Scheme 1:**
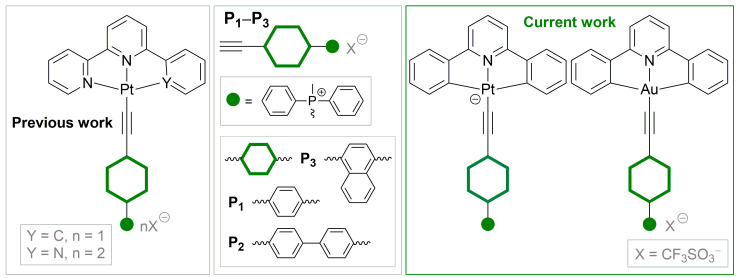
The alkynylphosphonium salts **P1**−**P3** used as ligands and the Pt(II) and Au(III) complexes based on them.

Despite the fact that Pt(II) and Au(III) are isoelectronic metallocentres, there is still no literature data regarding a comparison of their complexes directly. In this contribution, we synthesized and compared two bis-cyclometalated series of Au(III) and Pt(II) with CNC and alkynylphosphonium ligands (Scheme 1).

Previous studies of the Pt(II) complexes with CNN/NNN and alkynylphosphonium ligands revealed intriguing photophysical properties. Among others, the complexes [Pt(NNN)(C_2_−L−P(CH_3_)Ph_2_)]^2+^ in the solid state show significantly different emission wavelengths upon variation in anion size [13]. Herein, we seek to discover if alkynylphosphonium complexes of Pt(II) and Au(III) with ancillary CNC ligands can be called “analogues” in terms of their photophysical properties. Also, the influence of the charged PR_4_^+^ fragment on the complexes’ emission properties is considered.

## 2. Results and Discussion

### 2.1. Synthesis and Characterization of Pt(II) and Au(III) Complexes

**Scheme 2:**
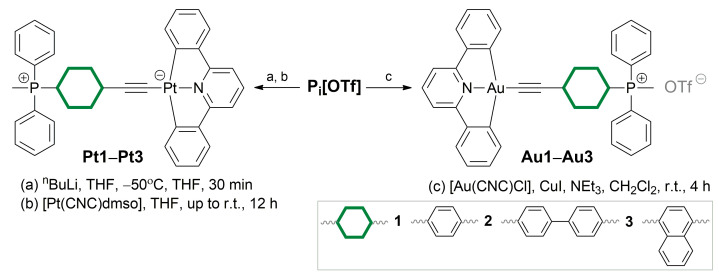
Synthesis of complexes **Pt1**–**Pt3** and **Au1**–**Au3**.

The Pt(II) complexes **Pt1**−**Pt3** (Scheme S1) were synthesized in two steps (Scheme 2). At first, lithiation of the corresponding alkynylphosphonium proligand was made, and after stirring for 30 min, [Pt(CNC)dmso] was added. Compared to the literature method [14], higher yields were generally observed. In addition, our method involved a smaller amount of the alkynylphosphonium ligand (1:8 molar ratio of Pt(II) precursor to the alkyne in the literature versus the 1:2 molar ratio in our method). This general procedure worked well for complexes **Pt1** and **Pt2**, whereas for **Pt3** it was not the case, with a reaction yield of less than 10%. We believe that it could be caused by some side reactions of the P_3_ ligand upon lithiation. Compounds **Pt1** and **Pt2** are sparingly soluble in acetone and acetonitrile and well-soluble in DMSO. Complex **Pt3** was insoluble and therefore was characterized only by FTIR (KBr pellets) and ESI^+^ mass-spectrometry.

The Au(III) complexes **Au1**−**Au3** (Scheme S1) were obtained by reaction of [Au(CNC)Cl] with the corresponding alkynylphosphonium salt under oxygen-free conditions in DCM in the presence of triethylamine and a catalytic amount of CuI (Scheme 2). Due to the presence of chloride ions in the starting compound, the reaction mixture contained complexes with two different counterions (Cl^−^ and OTf^−^) and was separated by column chromatography. Chloride-containing derivatives demonstrated lower yields, lower solubility and similar photophysical properties compared to the triflate ones, and thus they will not be considered in this work. All obtained Au(III) complexes are highly soluble in DMSO and DCM. However, **Au2** precipitates out of the DCM/acetone mixture, after which its solubility is significantly reduced.

The identity of all studied complexes was fully established by ^1^H, ^31^P{^1^H}, ^19^F{^1^H} and ^1^H^1^H COSY NMR experiments together with ESI^+^ mass-spectrometry and FTIR spectroscopy. In the ^1^H NMR spectra, which were interpreted using a ^1^H^1^H COSY experiment, all the integral intensities and multiplicities correspond to the proposed ones (Figures S1−S5). ^31^P{^1^H} NMR spectra of both series demonstrate one signal at ca. 21 ppm, which is characteristic for the phosphonium group (Figures S1−S5) [13,15,16,17]. ^19^F{^1^H} NMR spectra of complexes **Au1**–**Au3** exhibit only one singlet signal at 77.8 ppm, which is attributed to the triflate anion (Figures S3−S5).

The ESI^+^ mass spectra of **Pt1** and **Pt2** are dominated by two signals, corresponding to the [M + H]^+^ and [M + MeOH + H]^+^ fragments, as the studied compounds are neutral. In the **Pt3** ESI^+^ mass spectrum, the [M + H]^+^ fragment could also be found, but due to the extremely low solubility, the intensity of the signal is very low (Figure S6). The ESI^+^ mass spectra of cationic **Au1**−**Au3** show high-intensity signals corresponding to the [M]^+^ ion (Figure S7).

FTIR spectra of all complexes show the C≡C vibrations in the expected area (2000−2150 cm^−1^, Figure S8). It has been found that the C≡C stretching frequencies observed for the Au(III) complexes (2144−2152 cm^−1^) are very different from those found for the Pt(II) complexes (2068−2073 cm^−1^), and this notable difference can be explained by the following reasons. The observed higher ν(C≡C) values for the Au(III) complexes compared to their Pt(II) analogues can be rationalized by considering the differences in the electronic structures and bonding characteristics of the two metal centres. Pt(II) centre is generally more effective π-backdonor compared to Au(III), due to the higher covalence and lower effective nuclear charge of Pt(II). This allows Pt(II) to donate electron density from its filled d-orbitals into the π* orbital of the alkynyl ligand, thereby weakening the C≡C bond and resulting in a lower ν(C≡C) stretching frequency. In contrast, Au(III) is less capable of π-backdonation due to its higher oxidation state and greater relativistic stabilization of the 5d orbitals. As a result, the C≡C bond in Au(III) complexes retains more triple-bond character, leading to higher ν(C≡C) values. On the other hand, this difference can be discussed on the basis of bond strength estimates obtained from DFT calculations. The calculated frequencies for the C≡C bond are 2288 cm^−1^ (**Au1**), 2290 cm^−1^ (**Au2**), 2283 cm^−1^ (**Au3**), 2191 cm^−1^ (**Pt1**), 2202 cm^−1^ (**Pt2**), and 2183 cm^−1^ (**Pt3**), which are generally in line with the experimental IR spectra. The corresponding C≡C bond orders (computed as Wiberg bond indexes in the NAO basis) are equal to 2.75 (**Au1**), 2.76 (**Au2**), 2.74 (**Au3**), 2.64 (**Pt1**), 2.68 (**Pt2**), and 2.63 (**Pt3**). These values can be correlated with the **M−C**≡C bond orders, which are 0.73 for Au complexes, 0.84 for **Pt1** and **Pt3**, and 0.82 for **Pt2**. Thus, the bond between Pt and the carbon atom on the C≡C unit appears to be stronger than a similar bond with Au, which makes the triple bond weaker and, consequently, the frequencies for the Pt complexes are lower.

**Figure 1:**
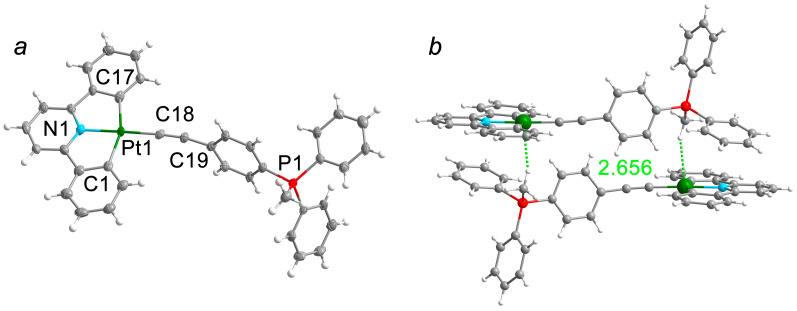
(**a**) ORTEP view of **Pt1** molecular structures, the thermal ellipsoids are set at a 50% probability level; (**b**) supramolecular dimer in the solid state of **Pt1** with CH···Pt short contacts formed by a CH_3_ group, H−Pt distances are indicated by the green dotted line. Solvent molecules are omitted for clarity.

Complex **Pt1** was crystallized from the diluted acetone solution. The key crystallographic details and some selected bond angles and distances are summarized in Tables S1 and S2. Single-crystal XRD studies revealed the expected distorted square-planar configuration of the metal centre (Figure 1a). Among the four Pt(II) bonds, the Pt–C_aryl_ distances are nearly the same, whereas Pt–N and Pt–C_alkynyl_ are much shorter. The bond angle between C_aryl_–Pt–C_aryl_ differs from the ideal 180° by 20°, which is caused by the rigidity of the CNC ligand. The {Pt−C≡C−R} fragment of the structure is slightly bent, and as we suppose, it can be induced by the bulky phosphonium fragment. Overall, the observed crystallographic parameters are in line with the reported experimental data of Pt(II) compounds with CNC ligand [14].

The packing of **Pt1** demonstrates paired molecules in head-to-tail fashion, which is easily explained by the zwitterionic nature of the compound (Figure 1b). These supramolecular dimers form two layers, arranged in a “herringbone” manner. Also, the Pt∙∙∙H intermolecular interaction has been found in the dimer of **Pt1** between the Pt atom and the hydrogen of the CH_3_ group with a distance of 2.565 Å and an angle Pt−H−C of 159°. This type of interaction can be considered as a rare case of hydrogen bond with Pt(II) atom as acceptor or attractive agostic interaction [18]. Such intermolecular interactions, such as Pt∙∙∙H−E, have been previously described for Pt(II) complexes with E = O, N [19,20,21]. In our case, the high Lewis basicity of the platinum atom is the result of CNC ligand coordination, and the mobility of the CH_3_ group proton is provided by the acceptor nature of the phosphonium group.

The Hirshfeld surface of **Pt1** was mapped over d_norm_ (range −0.1 Å to 1.5 Å). Red spots on the surface denote the dominant interactions (Figure S9). In our case, we aimed to prove the existence of the intermolecular interaction Pt−H, as well as to determine the contribution of this interaction to the total Hirshfeld surface area, so only this interaction is shown (Figure S9). The Hirshfeld surface analysis indeed shows a Pt−H contact where the hydrogen of the CH_3_ group of the phosphonium fragment interacts with the metal centre, and the contribution of this interaction is 2.5%. In addition, the shape index (Figure S9) shows a π-π interaction between the phenyl ring at the phosphorus atom and the CNC ligand, which may further stabilize the Pt−H bond.

### 2.2. Optical and Photophysical Properties

**Figure 2:**
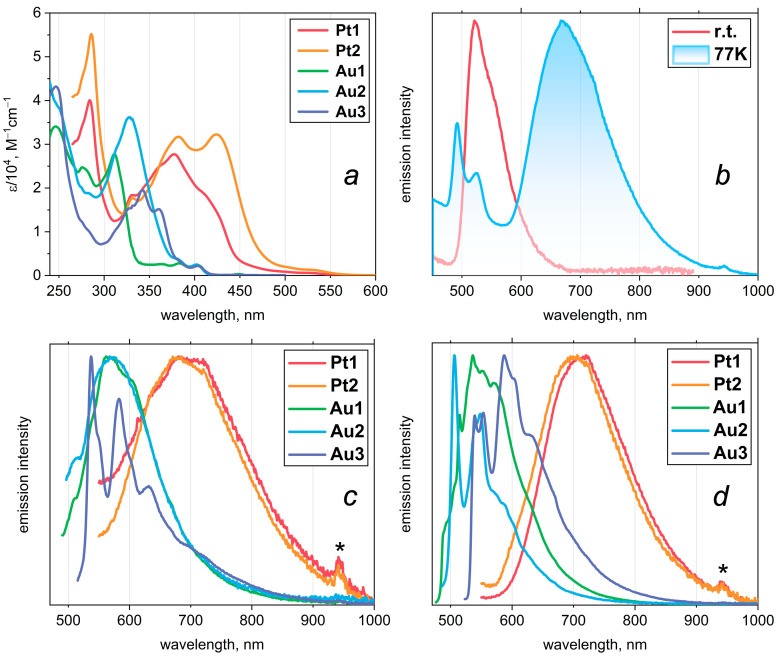
(**a**) UV-vis absorption spectra of **Pt1**, **Pt2 (**DMSO solution) and **Au1**–**Au3** (DCM solution), r.t. (**b**) Normalized emission spectra of **Pt1** in deaerated and frozen DMSO solution. (**c**,**d**) Normalized solid-state emission spectra of the **Pt1**, **Pt2** and **Au1**–**Au3** at (**c**) 292K and (**d**) 77K. An asterisk (*) denotes an instrument artefact.

The UV-Vis spectra of the complexes **Pt1** and **Pt2** were measured in DMSO solution due to low solubility. Absorption wavelengths are summarized in Table 1, and the corresponding spectra are depicted in Figure 2a.

Similarly to the literature data, the studied Pt(II) complexes exhibit strong absorption bands in high-energetic (<300 nm) and low-energetic (350–450 nm) parts of the spectrum [14,23,24,25,26,27,28]. With reference to the abovementioned studies, the former ones were assigned to the ^1^LC transitions, localized both at CNC and alkynylphosphonium ligands. Weaker red-shifted bands arise from the complex IL/LL’/MLCT transitions, namely mixed ^1^MLCT (dπ(Pt)→CNC) and ^1^LL’CT (CNC→alkynyl). We presume that the bands at 378 nm and 381 nm for **Pt1** and **Pt2**, respectively**,** have more ^1^MLCT character, as they are nearly independent of the alkynylphosphonium substituent. On the contrary, the absorption bands at ca. 410 nm (**Pt1**) and 423 nm (**Pt2**) can correspond to the different alkynylphosphonium ligand moiety. Thus, the extended π-conjugated linker in the ligand **P_2_** brings additional π-donating ability, which not only bathochromically shifts the corresponding absorption band but also enhances the extinction coefficient, as the ^1^LLCT becomes more efficient.

There is also an additional band in the 500–550 nm region of the UV-vis spectrum of both **Pt1** and **Pt2** (Figure S10). It could not be attributed to the d–d transitions because of the CNC ligands’ strong σ-donating nature. In addition, these transitions are unlikely to be observed at such low energies. Similar low-intensity red-shifted absorption bands are examined in the works by Che and Yam, and with this in mind, we ascribe this band to a spin-forbidden ^3^LC transition [23,24]. It becomes possible due to the large spin–orbit coupling, caused by the presence of the Pt atom as the metal centre.

The UV-Vis spectra of **Au1**–**Au3** in DCM demonstrate three absorption bands (Table 1, Figure 2a). The high-energetic bands ca. 250 nm, apparently, can be assigned to the ^1^LC transitions in alkynylphosphonium ligands. Then, the bands in the range 310−380 nm demonstrate a bathochromic shift associated with an increase in conjugation or condensation of linkers. This suggests localization of the transition on alkynylphosphonium ligands. In the case of **Au1** and **Au2**, the structureless shape of bands allows us to attribute the transitions to ILCT (alkynyl/linker→PR_4_^+^) state. At the same time, for **Au3** structured band with vibrational progression from 1260 to 1440 cm^−1^ testifies to ^1^LC transition localized on the naphthyl moiety, which was reported previously for complexes bearing the same alkynylphosphonium ligand [13,15,16,17]. Finally, the bands in the range of 375–425 nm are characteristic of [Au(CNC)C_2_R] and can be attributed to ^1^LC at CNC ligand mixed with ^1^ILCT (phenyl→pyridine ring) [6,29,30,31,32]. Interesting that the same bands for **Pt1** and **Pt2** are more red-shifted, which can be explained by a stronger spin–orbit coupling of the Pt atom relative to the Au one, or by the stronger electron-donating ability of Pt(II) compared to the Au(III).

**Figure 3:**
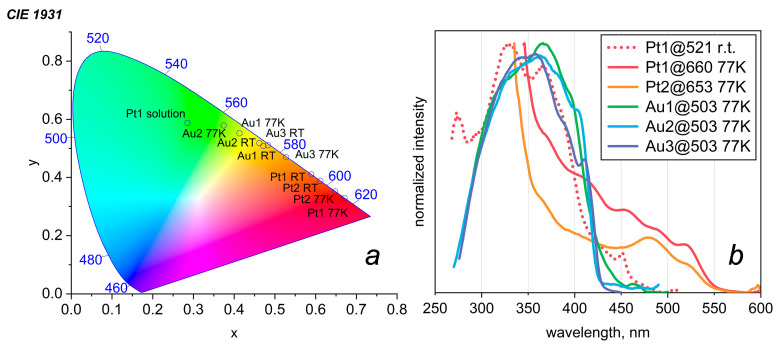
(**a**) CIE 1931 coordinates of **Pt1** (in deaerated DMSO solution, r.t.), and **Pt1**, **Pt2**, **Au1**−**Au3** (solid state, r.t. and 77 K). (**b**) Excitation spectra of **Pt1** (in DMSO solution, r.t.), and **Pt1**, **Pt2**, **Au1**−**Au3** (solid state, 77 K); emission wavelengths are indicated on diagram.

Upon excitation at 365 nm, deaerated DMSO solution of complex **Pt1** surprisingly demonstrates green emission (Table 1 and Table S3; Figure 2b and Figure 3a). This is in sharp contrast with the reported Pt(II) analogue without phosphonium fragment, [14] and that means that introduction of an electron-withdrawing group “turns on” the luminescence in this case. Based on the excited-state lifetime values and the rise in emission intensity upon deaeration, we state its phosphorescent nature. We assume that this emission is of mixed ^3^MLCT + ^3^LC nature, because upon freezing the solution, this band demonstrates a hypsochromic shift and becomes vibronically structured (Figure 2b). In addition, we see one more band in the red part of the spectrum, ca. 700 nm, apparently arising because of **Pt1** aggregated forms. In both cases, luminescence is not intensive, and for the **Pt1** solution, the value of emission quantum yield is less than 1% (Table 1). According to the literature [33], the absence of emission in [Pt(CNC)(alkynyl)] complexes has a fundamental nature: upon excitation, structural distortion takes place, paving the way for non-radiative relaxation. The emergence of luminescence in **Pt1** can presumably be caused by the introduction of an accepting phosphonium moiety on the periphery of the ligand environment. Thus, this fragment can withdraw the excess of electron density from the {Pt(CNC)} fragment and, by this means, hinder the structural distortion upon the MLCT excitation. Complex **Pt2** decomposes in solution under UV irradiation.

In contrast to the Pt(II) complexes, the studied Au(III) complexes demonstrate extremely low-intensity emission even in deaerated DCM or DMSO solution upon excitation at 365 nm. It may also be related to the presence of a strong electron-acceptor group on the periphery of alkynyl ligands. The cationic phosphonium fragment decreases the σ-donor properties of the alkynyl moiety and makes non-emissive d-d states of Au(III) accessible [34].

Complexes **Pt1** and **Pt2** show red luminescence in the solid state, both at room temperature and at 77K (Table 1 and Table S3; Figure 2c,d and Figure 3a). Their emission spectra at 298K show broad, structureless bands at ca. 700 nm. It is interesting to note that the spectra of the studied compounds are almost identical both at room and low temperature, thus implying a similar emission state. Upon cooling, the emission demonstrates a small bathochromic shift and intensifies, which is characteristic of the triplet character of luminescence. We assume that this emission could be attributed to either ^3^MC or “π-dimeric” luminescence, similar to **[Pt(CNN)P_1_]Cl** and **[Pt(NNN)P_2_]Cl**, discussed in our previous work [13]. In order to make the attribution more exact, we have compared the emission spectra and lifetime values with the literature example of similar complexes without a phosphonium fragment [14]. The emission spectra of **Pt1** and **Pt2** are more red-shifted compared to the NBu_4_[Pt(CNC)(C_2_Ph)], and the red shift upon cooling is less pronounced, coming mainly from the band narrowing. The solid-state excited state lifetime values for **Pt1** and **Pt2** are about ten nanoseconds, and the literature analogue demonstrates values three orders of magnitude greater. The authors of [14] state the ground state (or excimeric) ^3^ππ* excited states for NBu_4_[Pt(CNC)(C_2_Ph)], and depending on this fact, we attribute our emission to the ^3^MC nature. The excitation spectrum profile (Figure 3b) also supports this version of the Pt(II) complexes’ emission nature in the solid state. All of the abovementioned facts mean that, in contrast with the emission in solution, the alkynylphosphonium ligand does not participate in the solid-state luminescence of **Pt1** and **Pt2** directly.

The complexes **Au1**–**Au3** demonstrate a yellow to orange luminescence in the solid state (Table 1 and Table S3; Figure 2c,d and Figure 3a). However, as in the solution, this emission has extremely low intensity at room temperature. The complexes **Au1** and **Au2** demonstrate an emission band with maxima at ca. 560 nm at room temperature. Cooling of the complexes to 77K increases the luminescence efficiency, which, together with the long lifetime of the excited state, indicates the triplet nature of emission. Upon cooling, a hypsochromic emission shift accompanied by a colour change is observed for both complexes (Table 1 and Table S3; Figure 2c,d and Figure 3a). The **Au1** demonstrates an emission band with a weakly pronounced structure. Grinding of the fine crystalline powder, as well as cooling, does not essentially change the shape of the band. This suggests that the emission is characterized by a metal-perturbed ^3^LC at CNC ligand with admixture of ^3^MLCT (metal→CNC or metal→alkynyl) or ^3^ILCT at alkynylphosphonium ligand.

In the case of **Au2**, a structureless band has been observed upon photoexcitation. However, after grinding, the shape of the band changes (Figure S11). Cooling to 77K leads to an even more pronounced vibronic progression of this band (ca. 1250 cm^−1^). This can be explained as a coexistence of low-lying emissive states ^3^MLCT and ^3^LC, with the larger contribution of the former in the case of room-temperature **Au2** emission. Since we consider the solid phase, it is possible that ^3^LC radiative relaxation is blocked due to aggregation-induced quenching. Grinding the sample partially disrupts aggregation, resulting in two pathways of luminescence. Finally, upon cooling, we observe “temperature switching” of emissive states, as described in the literature [16,35]. The emission spectrum for **Au3** contains one band with a vibronic progression of 1450 cm^−1^. There is no shift in the band upon cooling. This, as well as the long lifetime of the excited state, allows us to attribute the emission for **Au3** to metal-perturbed ^3^LC localized at the naphthyl moiety.

The photophysical properties of Au(III) complexes containing alkynylphosphonium auxiliary ligands were compared with those of complexes containing alkynylammonium ligands studied by Yam’s group [36]. These complexes were shown to exhibit low quantum yields in both solution and solid phase, with a maximum reported value of 1%. In both cases, this phenomenon can be explained by a decrease in the σ-properties of the auxiliary ligand, which consequently leads to a decrease in the efficiency of d-d splitting. This effect is particularly pronounced in the **1-OTf** complex, which has the closest structural similarity to **Au1**−**Au3** and does not exhibit luminescence in the solid state at room temperature. This is probably due to the enhanced acceptor properties of the trimethylammonium moiety. It is interesting to note that the excited state lifetime of **Au1**−**Au3** in the solid state at room temperature is much longer than that of other alkynyl bis-cyclometalated Au(III) complexes reported in the literature [36,37,38]. This phenomenon can be explained by the increased contribution of the metal to the excited state due to the presence of a strong acceptor in the ligand environment. This, in turn, facilitates the realization of the ^3^MLCT(metal→alkynyl) state.

### 2.3. DFT Calculations

**Figure 4:**
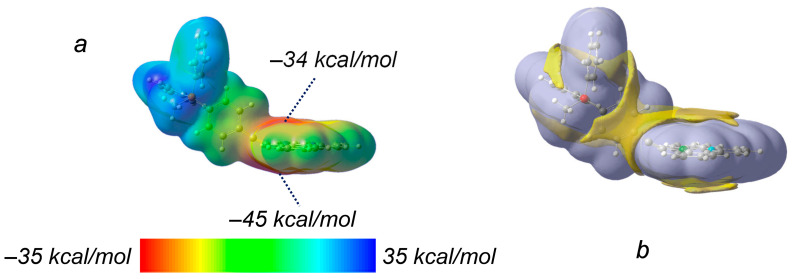
(**a**) Electron density isosurface (0.001 a.u.) coloured by molecular electrostatic potential (MESP) for the equilibrated **Pt1** monomer. MESP local maxima values near the Pt atom are indicated. (**b**) Van der Waals potential isosurface of the interaction energy between the **Pt1** monomer and a He atom, equal to ±0.5 kcal/mol. The grey region corresponds to the exchange-repulsion term, and the yellow regions to the dispersion term.

The crystal structure of **Pt1** revealed a unique CH···Pt interaction. To further investigate the nature of this interaction, we performed computational modelling. Analysis of the equilibrium geometry of the **Pt1** monomer reveals an anisotropic distribution of the molecular electrostatic potential (MESP) around the platinum atom (Figure 4a). The MESP is lower (approximately –34 kcal/mol) on one side of the platinum atom and higher (around –45 kcal/mol) on the opposite side, indicating a polarization of electron density. The van der Waals potential map shows regions near the platinum atom that are favourable for dispersion interactions (Figure 4b). These observations suggest that the platinum atom in **Pt1** is capable of participating in non-covalent interactions with electron density acceptors through both electrostatic and dispersion forces.

**Figure 5:**
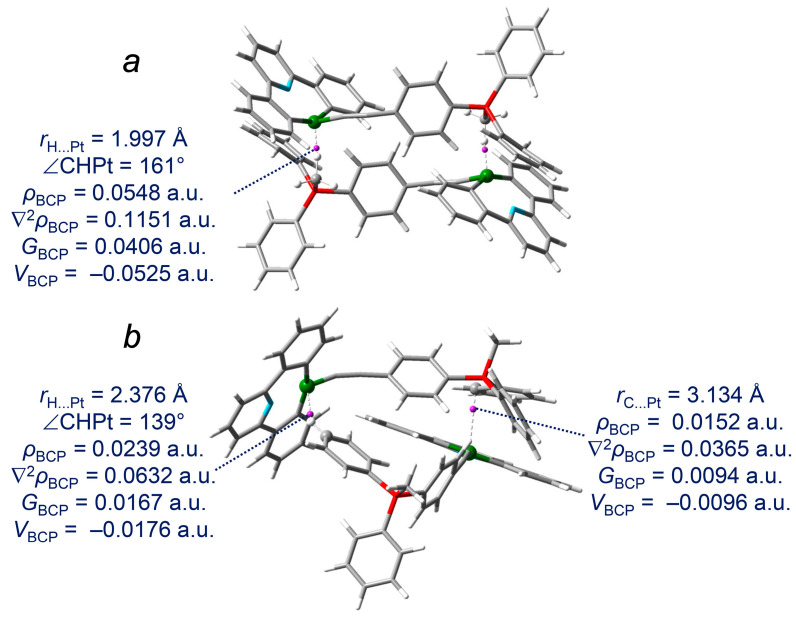
Optimized geometries of **Pt1** supramolecular dimers with CH···Pt short contacts formed by (**a**) a CH_3_ group and (**b**) a CH proton of the phenyl ring acting as the proton donor. The dashed lines illustrate the bond paths between (3; −1) and (3; −3) electron density critical points that involve platinum (green), nitrogen (blue), and carbon (black) atoms. (3; −1) critical points are given in violet. Corresponding geometric parameters (interatomic distance *r*_H…Pt_, *r*_C…Pt_ and angle CHPt) and electron density parameters in (3; −1) critical points (electron density *ρ*, the Laplacian of electron density ∇^2^ρ, density of local electronic kinetic *G* and potential *V* energies) are given in dark blue.

To assess the stability of potential CH···Pt interactions, we optimized the geometries of Pt1 supramolecular dimers with short CH···Pt contacts, where CH groups of the methyl group or the phenyl ring act as proton donors (Figure 5). During geometry optimization, both CH···Pt contacts shorten, and the structural framework of each monomer undergoes notable changes. For the dimer involving the CH proton of the methyl group, optimization leads to the formation of a second, equivalent CH···Pt bond with the methyl proton of the phosphonium group of the initially accepting molecule. Analysis of the electron density parameters in the optimized dimer structure (Figure 5a) suggests that this CH···Pt interaction has a moderate strength, estimated to be approximately 10 kcal/mol. This suggests that this type of CH···Pt interaction is energetically favourable and not solely a consequence of crystal packing. It is important to note that this value represents an upper estimate of the actual interaction strength in the crystal. Furthermore, NBO analysis reveals a noticeable charge transfer from the hydrogen atom to the platinum atom, further supporting the stabilizing nature of this interaction.

For the second dimer, optimization also results in a shorter interatomic distance between the hydrogen and platinum atoms. However, the C−H−Pt angle is significantly distorted in the optimized structure (139°). This indicates that this type of CH···Pt interaction is less likely to be a dominant structure-forming factor, suggesting the presence of other, more energetically favourable interactions in the crystal. For instance, the distortion observed in the optimized dimer structure in Figure 5b correlates with the formation of Pt···π interactions.

In summary, computational modelling focused on **Pt1** revealed the significance of non-covalent interactions, particularly CH···Pt, in influencing the supramolecular assembly. While Pt···π interactions may also play a role, the CH···Pt interactions are demonstrated to be energetically favourable, potentially contributing to the solid-state packing observed in the crystal structure. The observed anisotropic distribution of the molecular electrostatic potential around the Pt atom suggests its ability to engage in both electrostatic and dispersion interactions with neighbouring molecules. Despite the relatively short H···Pt distances, the positive Laplacian of the electron density at the bond critical point suggests that these interactions are best described as hydrogen bonds, rather than agostic interactions. Agostic interactions, commonly observed in transition metal complexes, involve the coordination of a C−H σ-bond to a metal centre, typically characterized by a negative Laplacian of the electron density, indicating a concentration of electron density between the metal and hydrogen atoms [39,40,41]. These findings emphasize the importance of considering non-covalent interactions in understanding the photophysical properties and solid-state behaviour of this class of alkynylphosphonium Pt(II) complexes.

In order to understand the origins of the observed photophysical behaviour of the studied compounds, quantum chemical calculations were carried out. The structures of **Pt1**−**Pt3** and **Au1**–**Au3** complexes were optimized by DFT calculations (Figures S12 and S13). The coordination environment of the platinum and gold atoms in complexes was found to be planar, and the N1−M−C18≡C19 fragments were found to be linear (M = Au, Pt; atom identifiers are similar to those used in Figure 1a). The main bond angles and distances are equal in complexes with the same coordination centre and slightly differ for various metals, namely, for bond lengths: M−N1 = 2.026/2.039 Å, M−C18 = 1.970/1.957 Å, M−C1(C17) = 2.085/2.081 Å, C18−C19 = 1.209/1.222 Å, angles: N1−M−C1(C17) = 80.65°/79.98°, C1(C17)−M−C18 = 99.40°/99.77°, C1−M−C17 = 161.30°/159.93°. For the **Pt1** complex, these values can be compared with the experimental ones. It can be seen from Table S2 and the values provided above that the difference between experimentally measured and calculated bond lengths and angles is less than 0.03Å and 1°, respectively, which suggests a good agreement of the structures obtained.

For **Au1**−**Au3** complexes, the main absorption band wavelengths, which are related to the active singlet transitions, with oscillator strengths *f* were obtained by TDDFT calculations; the values are 278 nm (*f* = 1.37), 294 nm (*f* = 1.77), and 323 nm (*f* = 0.80), respectively. While calculated wavelengths are blue-shifted by ca. 30 nm, as compared to the experimental UV-vis spectra, the effect of the linker on photophysical properties is exactly the same as in the experimental study: from **Au1** to **Au3** a bathochromic shift is observed; the oscillator strength (intensity) is the highest for **Au2** and the lowest for **Au3**.

**Figure 6:**
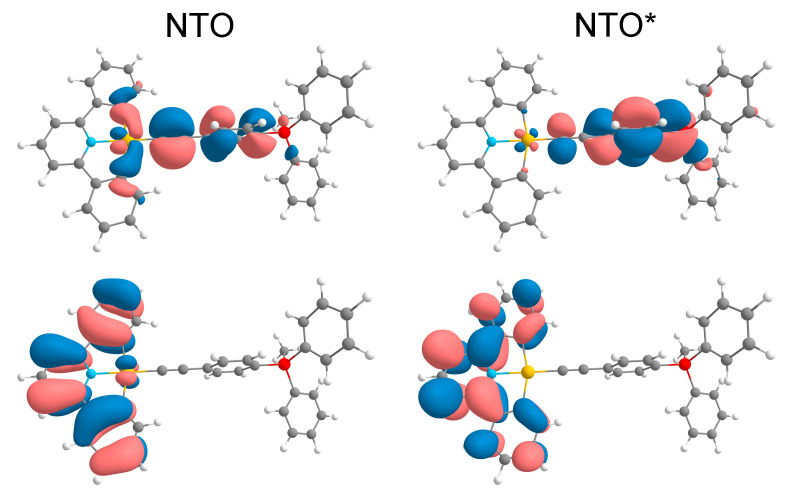
Natural transition orbitals for the active singlet S_3_^*^ of the complex **Au1** (278 nm, *f* = 1.37). The two main components of the transition are shown.

NTO analysis of TDDFT results shows that the nature of the active singlet excited state S_3_^*^ of **Au1** includes almost the entire complex: transition occurs from the gold atom + alkynyl ligand to the alkynyl ligand with a moderate contribution of the phosphonium fragment; the electron density is also redistributed within the pyridyl moiety, including the gold atom (Figure 6). To summarize the above, the transition in question can be characterized as a complex ILCT(alkynyl linker)/LC(CNC ligand)/LL’/MLCT one. For **Au2** and **Au3**, the active singlet excited states (S_3_^*^ and S_2_^*^, respectively) can be interpreted as ILCT/LL’/MLCT, while the transition occurs mainly within the central part of the complexes, and the contribution of CNC ligand is minimal (Figures S14 and S15).

The lowest singlet states for **Au1**−**Au3** complexes with a calculated wavelength of 336 nm are of the same nature and can be described as IL transitions within the pyridyl moiety with an admixture of the metal (Figures S16−S18).

**Figure 7:**
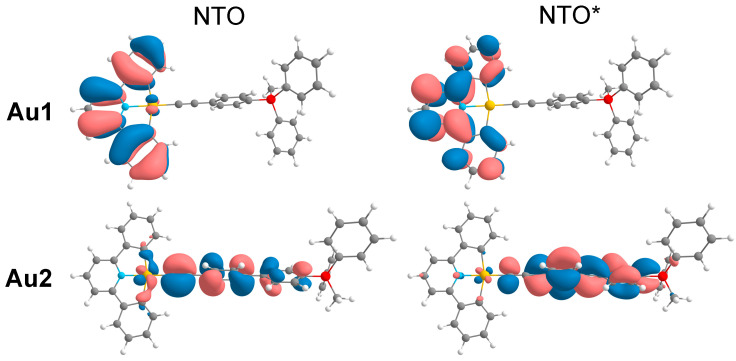
Natural transition orbitals for the lowest triplet T_1_ of the complexes **Au1** (442 nm) and **Au2** (443 nm).

The lowest triplet state T_1_ (Figure 7) for the **Au1** complex with a calculated wavelength of 442 nm primarily involves the pyridyl ring of the CNC ligand and can be characterized as an intraligand with an MLCT contribution. A different nature is observed for the lowest triplet transitions in **Au2** and **Au3** complexes (443 nm and 539 nm, respectively). The transitions resemble the active singlets and occur mainly inside the alkynyl linker with a moderate participation of gold atom (Figure 7 and Figure S19). Even though the lowest triplet transitions for **Au1** and **Au2**/**Au3** are of different nature, all of them can be characterized as LC with MLCT admixture.

The results obtained can be compared with the results of the recent study of very similar gold complexes with the phosphine oxide group instead of the phosphonium fragment [42]. The values of wavelengths of active singlets, lowest singlets, and triplets appear to be very close for the related gold complexes with the same linker, but the nature of the same transitions may be different in some cases. The most pronounced difference is observed for the nature of **Au1** active singlet and **Au2** lowest triplet transitions. In the present study, the lowest active singlet S_3_^*^ transition for **Au1** complex engages almost the whole molecule, while in the case of [Au(CNC)C_2_−L−P(O)Ph_2_] only the CNC ligand and the metal atom take part in the transition. For the triplet T_1_ transition in **Au2**, it was found that the redistribution of electron density occurs mainly within the alkynyl linker, while in the related complex [Au(CNC)C_2_−L−P(O)Ph_2_], this transition affects only the pyridine fragment.

For **Pt1** and **Pt2** complexes, TDDFT calculations for the optimized ground states yield controversial results. For this reason, computational data for these two complexes were obtained via the optimization of the lowest triplet structure. **Pt3** was not considered due to the unavailability of the corresponding experimental data.

The main absorption band wavelengths for **Pt1** and **Pt2** complexes obtained by TDDFT are red-shifted compared to the **Au1** and **Au2** complexes. For both **Pt1** and **Pt2** complexes, low-energy active singlet transitions are observed: at 473 nm for **Pt2** with a high intensity (*f* = 2.0668) and at 449 nm for **Pt1** with a low intensity (*f* = 0.1123). These singlet transitions are the lowest ones. The S_2_ transition (409 nm) for **Pt1** is also active and has even higher intensity (*f* = 1.3656) than S_1_^*^. For **Pt2**, the second active singlet is S_3_ at 382 nm (*f* = 0.1551). These observations are in line with the experimental UV-vis spectra (Figure 2a).

The nature of excited states was analyzed, and the results show that for **Pt1** complex the active singlet transitions S_1_^*^ and S_2_^*^ resemble the S_3_^*^ transition for **Au1** complex, i.e., involve almost all parts of the molecule (Figure S20): the electron density is redistributed between the alkynyl ligand, metal atom and pyridine fragment. The active singlet transition S_1_^*^ for **Pt2** occurs from the {linker + M + CNC(partly)} to the alkynyl fragment (Figure S21), which is also the case for the active singlet transition for the **Au2** complex. For the S_3_^*^ transition (the second active singlet), it can be seen that electron density is transferred from {C≡C + M + CNC} fragment almost entirely to the pyridine moiety.

Since the optimization of the lowest triplet transitions for **Pt1** and **Pt2** was provided, the structures obtained can be compared with the optimized structures of the ground states. For **Pt1**, the most pronounced difference is in the dihedral angle between the plane of the pyridine fragment and the phenyl ring of the alkynyl linker (Figure S22). For the ground state, this dihedral is 92.6°, for the triplet T_1_ state, the value is 161.0°, i.e., the orientation of the planes changes from almost perpendicular to the near-parallel. For **Pt2**, the reorientation of phenyl rings is observed in the T_1_ triplet state (Figure S23). The value of the dihedral angle between the rings changes from 143° to 180°.

**Figure 8:**
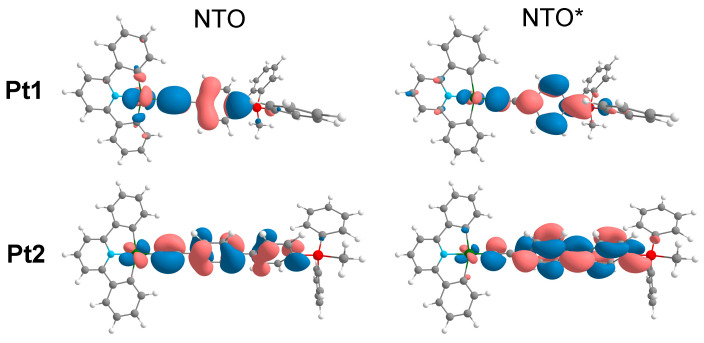
Natural transition orbitals for the lowest triplet T_1_ of the complexes **Pt1** (661 nm) and **Pt2** (840 nm).

The lowest triplet state wavelength of **Pt1**, obtained by DTDFT calculations, is 661 nm. This transition occurs within the alkynyl ligand with the moderate participation of the platinum atom, suggesting a mixed LC/MLCT assignment (Figure 8), which is completely different from the transition in the **Au1** complex with the same ligand. The possible cause of this difference could be the different orientation of the phenyl ring in the alkynyl linker. The **Pt2** complex has a triplet state with a very low energy (840 nm), which cannot be realized in the solution due to complex destruction. The nature of the transition is similar to that observed for **Pt1** and **Au2** complexes (Figure 8).

Based on the NTO analysis, the contributions of the metal to the triplet transitions can be calculated. It was found that the Au atom contribution is negligible (about 1%) for both **Au1** and **Au2** complexes. The Pt atom participation in triplet transitions is more pronounced: 9% (NTO) and 5% (NTO*) for **Pt1** complex, 4% (NTO) and 2% (NTO*) for **Pt2** complex. Thus, while the contribution of platinum to T_1_ transition is quite low, it is still more significant than that for gold.

Comparing the series of Au and Pt complexes in general, it could be mentioned that for **Pt1** complex the lowest active singlet S_1_^*^ is the fifth lowest state in the calculated spectrum, for **Pt2** S_1_^*^ — the second lowest, while for **Au1**, **Au2**, and **Au3** complexes the lowest active singlets are the 19th, 15th, and 10th states in spectra, respectively. As a result, Au(III) complexes have significantly more opportunities for non-radiative transitions and thus demonstrate extremely low intensity emission in the experimental measurements.

## 3. Materials and Methods

Precursory phosphonium salts **[P_i_]OTf** [15], [Pt(CNC)dmso] [43] and [Au(CNC)Cl] [44] were synthesized according to the literature methods. All other reagents were purchased from Merck, Alfa Aesar, and ABCR and used without further purification. Solvents were purchased from Vecton and purified according to the published methods [45]. All Pt(II) and Au(III) complexes discussed were obtained under an inert atmosphere using Schlenk equipment. The characterization included NMR spectroscopy (Bruker Avance Neo, Avance III), mass spectrometry (Maxis Bruker Daltonic, ESI+ mode), and FTIR spectroscopy (Shimadzu IRAffinity).

**Synthesis of Pt(II) complexes.** The synthesis of complexes **Pt1**–**Pt3** was carried out according to the modified literature method [14]. At the first step, 0.28 mmol (2 eq) of corresponding alkynylphosphonium salt **[P_i_]OTf** was dissolved in 10 mL of freshly distilled THF, degassed and cooled to –50 °C. Then, 10% excess of 2.5M ^n^BuLi hexanes solution was added (ca. 0.1 mL). The resulting yellow solution was stirred for half an hour. After that, 0.14 mmol (1 eq) of [Pt(CNC)dmso] was added under argon and the reaction mixture was left to stir overnight, slowly coming to room temperature. The resulting suspension was centrifuged. The orange precipitate was washed with DCM (4 times), methanol (1 time), diethyl ether (1 time) and dried in vacuo.

**[Pt(CNC)(P_1_)], Pt1.** Orange powder, yield 56%. ^1^H NMR (400 MHz, DMSO-*d*_6_) δ 7.87 (m, 2H, P_1_), 7.77 (m, 8H, P_1_), 7.76 (m, 2H, CNC), 7.66 (t, *J* = 7.9 Hz, 1H, CNC), 7.50 (m, 6H, P_1_), 7.49 (m, 2H, CNC), 7.03 (m, 2H, CNC), 6.92 (m, 2H, CNC), 3.06 (d, *J* = 14.3 Hz, 3H, P_1_). ^31^P{^1^H} NMR (162 MHz, DMSO-*d*_6_) δ 21.74 (s, 1P, P_1_). ESI HRMS (*m*/*z*): calcd for [M + H]^+^: 725.1687; found: 725.1689. FTIR (KBr): 2067 cm^−1^ (C≡C vibration). Single crystals of **Pt1** were obtained by the slow evaporation of diluted acetone solution.

**[Pt(CNC)(P_2_)], Pt2.** Orange powder, yield 66%. ^1^H NMR (400 MHz, DMSO-*d*_6_) δ 8.07 (dd, *J* = 8.5, 2.8 Hz, 2H, P_2_), 7.90 (m, 2H, P_2_), 7.84 (m, 2H, CNC), 7.77 (m, 10H, P_2_), 7.70 (d, *J* = 8.5 Hz, 2H, P_2_), 7.65 (t, *J* = 7.9 Hz, 1H, CNC), 7.48 (d, *J* = 7.9 Hz, 2H, CNC), 7.38 (d, *J* = 24.3, 8.4 Hz, 4H, P_2_), 7.06 (m, 2H, CNC), 6.93 (m, 2H, CNC), 3.13 (s, 3H, P_2_). ^31^P{^1^H} NMR (162 MHz, DMSO-*d*_6_) δ 22.21 (s, 1P, P_2_). ESI HRMS (m/z): calcd for [M + H]^+^: 801.2004; found: 801.2001. FTIR (KBr): 2072 cm^−1^ (C≡C vibration).

**[Pt(CNC)(P_3_)], Pt3.** Dark-brown powder, yield 8%. ESI HRMS (*m*/*z*): calcd for [M + H]^+^: 775.1845; found: 775.1840. FTIR (KBr): 2053 cm^−1^ (C≡C vibration).

**Synthesis of Au(III) complexes.** The synthesis of complexes **Au1–Au3** were carried out according to the modified literature method [29]. The solid residue was purified by column chromatography on silica using dichloromethane/acetone (5/1→5/3 *v*/*v*) as eluent.

**[Au(CNC)(P_1_)]OTf, Au1.** Beige powder, yield 94%. ^1^H NMR (400 MHz, DMSO-*d*_6_) δ 8.18 (t, *J* = 7.95 Hz, 1H, CNC), 7.98 (d, *J* = 8.0 Hz, 2H, CNC), 7.95–7.67 (m, 18H, CNC/P_1_), 7.40 (td, *J* = 7.3, 1.4 Hz, 2H, CNC), 7.34 (td, *J* = 7.5, 1.4 Hz, 2H, CNC), 3.15 (d, *J* = 14.5 Hz, 3H, P_1_). ^31^P{^1^H} NMR (162 MHz, DMSO-*d*_6_) δ 22.57 (s, 1P, P_1_). ^19^F NMR (376 MHz, DMSO-*d*_6_) δ −77.73 (s, 3F, OTf). ESI HRMS (m/z): calcd for [M]^+^: 726.1619; found: 726.1624. FTIR (KBr): 2152 cm^−1^ (C≡C vibration).

**[Au(CNC)(P_2_)]OTf, Au2.** Yellow powder, yield 65%. ^1^H NMR (400 MHz, DMSO-*d*_6_) δ 8.19 (t, *J* = 8.0 Hz, 1H, CNC), 8.15–8.07 (m, 2H, P_2_), 7.99 (d, *J* = 8.0 Hz, 2H, CNC), 7.92 (m, 6H, CNC/P_2_), 7.88–7.74 (m, 12H, P_2_), 7.73–7.65 (m, 2H, P_2_), 7.44 (td, *J* = 7.3, 1.3 Hz, 2H, CNC), 7.35 (td, *J* = 7.6, 1.4 Hz, 2H, CNC), 3.17 (d, *J* = 14.6 Hz, 3H, P_2_). ^31^P{^1^H} NMR (162 MHz, DMSO-*d*_6_) δ 22.43 (s, 1P, P_2_). ^19^F NMR (376 MHz, DMSO-*d*_6_) δ −77.73 (s, 3F, OTf). ESI HRMS (m/z): calcd for [M]^+^: 802.1938; found: 802.1925. FTIR (KBr): 2146 cm^−1^ (C≡C vibration).

**[Au(CNC)(P_3_)]OTf, Au3.** Beige powder, yield 80%. ^1^H NMR (400 MHz, DMSO-*d*_6_) δ 8.77 (d, *J* = 8.5 Hz, 1H, P_3_), 8.20 (t, *J* = 8.0 Hz, 1H, CNC), 8.08–7.97 (m, 3H, CNC/P_3_), 7.97–7.82 (m, 11H, CNC/P_3_), 7.82–7.74 (m, 5H, P_3_), 7.68–7.52 (m, 2H, P_3_), 7.41 (td, *J* = 7.3, 1.4 Hz, 2H, CNC), 7.35 (td, *J* = 7.5, 1.5 Hz, 2H, CNC), 3.28 (d, *J* = 14.0 Hz, 3H, P_3_). ^31^P{^1^H} NMR (162 MHz, DMSO-*d*_6_) δ 22.13 (s, 1P, P_3_). ^19^F NMR (376 MHz, DMSO-*d*_6_) δ −77.73 (s, 3F, OTf). ESI HRMS (m/z): calcd for [M]^+^: 776.1767; found: 776.1781. FTIR (KBr): 2144 cm^−1^ (C≡C vibration).

**X-Ray structure determination.** The details of the X-Ray structure determination can be found in the Supplementary Information. References [46,47,48,49,50] are cited there.

**Pt1**: I2/c (15), a = 24.9558(8), b = 12.2843(3), c = 21.9034(7) Å; α = 90, β = 115.373(4), γ = 90 °; *V* = 6067.1(4) Å^3^; *Z* = 4; *R*_1_ = 0.0482; CCDC 2383597.

**Hirshfeld surface analysis.** The intermolecular interactions in the crystal were quantified using Hirshfeld surfaces and the associated 2D fingerprint plot, which were explored by means of Crystal Explorer based on a structure input file from CIF (Crystallographic Information File) [51].

**Photophysical study.** The UV-vis absorption spectra were registered on a Shimadzu UV-1800 spectrophotometer in a 1 cm quartz cuvette (DMSO, DCM, 10^‒5^ M). The emission spectra for solid samples were recorded using an Avantes AvaSpec-2048×64 spectrometer (Avantes, Apeldoorn, The Netherlands). The pulse laser DTL-399QT ‘Laser-export Co. Ltd.’ (wavelength 351 nm, pulse energy 50 μJ, pulse width 6 ns, repetition rate 0.01−1 kHz), an Ocean Optics monochromator Monoscan-2000 (interval of wavelengths 1 nm; Ocean Optics, Largo, FL, USA), photon counting head H10682 (Hamamatsu), and multiple-event time digitizer P7887 (FAST ComTec GmbH) were used for solid-state lifetime measurements. The Avantes integration sphere (Avantes, Apeldoorn, The Netherlands) was used to measure the solid-state emission quantum yield.

**Computational details**

Theoretical investigation of complexes was provided by density functional theory (DFT) calculations. Calculations were performed using the Gaussian16 software (Revision A.03) package [52]. Geometry optimizations and harmonic vibrational frequency calculations were carried out, and all structures were verified to ensure the absence of imaginary frequencies.

In DFT calculations of photophysical properties, all complexes were considered using CAM-B3LYP range-separated hybrid functional [53]. The Au and Pt atoms were represented by SDD basis set with MWB60 effective core potential [54]; other atoms were described by the def2-TZVP basis set [55]. Calculations for complexes were performed in the presence of the implicit solvents (DCM for Au complexes and DMSO for Pt complexes), which were described by the C-PCM model [56,57]. Time-dependent DFT (TDDFT) calculations were carried out to investigate the excited states of complexes: for Au complexes, the optimized ground-state molecular geometries were used, while for Pt complexes, optimization of the excited states was required. The nature of the excited states was demonstrated via natural transition orbitals (NTO) [58]. For Pt–H bond modelling B3LYP-G3BJ/jorge-TZP level of theory was used [59,60]. The MultiWFN programme was employed for NTO analysis, electron density topology analysis and for calculating the surfaces of electron density, molecular electrostatic potential, and van der Waals potential [61,62,63]. Natural Bond Orbital (NBO) analysis was conducted using the NBO 7.0 programme [64]. Visualization was performed using GaussView 6.0 [65].

## 4. Conclusions

In this work, two series of Au(III) and Pt(II) alkynylphosphonium complexes with ancillary CNC ligand were synthesized and fully characterized. Complex **Pt1** possesses a Pt–H interaction in its crystal structure, confirmed by quantum-chemical calculations. This compound is also the first example of room-temperature solution luminescence in a family of [Pt(CNC)(alkynyl)] complexes. However, a short lifetime does suggest the presence of an effective non-radiative pathway of relaxation. These relaxation channels can be associated with an involvement of d-d states and with structural distortion, but they are less effective than for [Pt(CNC)(alkynyl)] analogues without an electron-withdrawing group in the alkynyl ligand. Au(III) complexes do not emit in the solution.

In the solid state, both series of complexes demonstrate weak emission. In the case of **Pt1** and **Pt2**, the spectra look almost identical and are independent of the alkynyl ligand. Moreover, the lifetime values are very small, and the bands narrow upon cooling. These facts indicate the ^3^MC emission nature. Turning to the Au(III) series, these complexes demonstrate emission with significant participation of the ligand. For the complexes with π-conjugated linkers, **Au1** and **Au2**, ^3^ILCT and ^3^LC, located at the CNC ligand, excited states are very close in energy, and can be switched upon cooling. For the complex with naphthyl linker **Au3**, the fused moiety plays a determinative role: only ^3^LC luminescence is observed.

To sum it up, Au(III) and Pt(II) bis-cyclometalated alkynylphosphonium complexes demonstrate different photophysical properties despite isoelectronic metal centres. Apparently, the oxidation state of the metal centre plays a considerable role. In the case of electron-deficient Au(III), the introduction of the acceptor to the periphery of the ligand environment additionally increases the metal centre electrophilicity and lowers the d-d states. All these factors worsen the emission properties of **Au1**–**Au3** complexes. On the contrary, withdrawing of excess electron density from the {Pt(CNC)} core with the help of accepting the phosphonium fragment allows for the stabilization of the structures of **Pt1** and **Pt2** upon photoexcitation, and thus enhances the photophysical properties.

## Figures and Tables

**Table 1:** Optical and photophysical properties of **Pt1**–**Pt2** and **Au1**–**Au3** complexes.

Complex	λ_abs_, nm	λ_em_, * nm	λ_em_, * nm		τ, ** ns	
		DMSO r.t.	Solid 77K	Solid r.t.	DMSO r.t.	Solid r.t.
**Pt1**	284, 332, 378, ~410, ~522	521	710	692	89 (aer) 147 (deg)	17
**Pt2**	286, 332, 381, 423, ~525	–	700	680	–	23
**Au1**	247, 276, 312, 368, 384, 403	–	536	563	–	55,218
**Au2**	250^sh,^ 275^sh^, 283, 328, 381^sh^, 402,	–	506	563	–	33,221
**Au3**	247, 278^sh^, 283^sh^, 328^sh^, 343, 360, 385^sh^, 403	–	587	538	–	14,958

* λ_exct_ 365 nm. ** λ_exct_ 351 nm. The amplitude average lifetime *τ^aver^* = ∑*A_i_τ_i_* was calculated according to the published method [22]. The values of emission quantum yield for all compounds are less than 1%.

## Data Availability

All data reported herein are accompanying the present article.

## Supplementary Materials

The following supporting information can be downloaded at https://www.mdpi.com/article/10.3390/molecules30112434/s1, Scheme S1: The complexes Pt1–Pt3 and Au1–Au3; XRD structure determination; Table S1: Crystallographic data for the compound Pt1; Table S2: Selected bond angles and distances for the compound Pt1; Figure S1: (A) ^1^H (top) and ^31^P{H} (bottom) spectra in aromatic range of Pt1; (B) ^1^H^1^H COSY spectra in aromatic range of Pt1; Figure S2: (A) ^1^H (top) and ^31^P{H} (bottom) spectra in aromatic range of Pt2; (B) ^1^H^1^H COSY spectra in aromatic range of Pt2; Figure S3: (A) ^1^H (top), ^31^P{H} (middle) and ^19^F (bottom) spectra in aromatic range of Au1; (B) ^1^H^1^H COSY spectra in aromatic range of Au1; Figure S4: (A) ^1^H (top), ^31^P{H} (middle) and ^19^F (bottom) spectra in aromatic range of Au2; (B) ^1^H^1^H COSY spectra in aromatic range of Au2; Figure S5: (A) ^1^H (top), ^31^P{H} (middle) and ^19^F (bottom) spectra in aromatic range of Au3; (B) ^1^H^1^H COSY spectra in aromatic range of Au3; Figure S6: Experimental (left) ESI^+^ MS spectra of Pt1–Pt3 and simulated (right) isotopic patterns of the [M+H]^+^; Figure S7: Experimental (left) ESI^+^ MS spectra of Au1–Au3 and simulated (right) isotopic patterns of the [M]^+^; Figure S8: FTIR spectra of Pt1–Pt3 and Au1–Au3 in the region of C≡C vibration, KBr; Figure S9: (a) Hirshfeld surface 2D fingerprint plot (all interactions), (b) Hirshfeld surface 2D fingerprint plot (Pt–H interaction), (c) d_norm_ Hirshfeld surface (Pt–H contacts only), and (d) shape index, Pt1; Figure S10: (a) The low-energy part of the UV-vis spectra of Pt1 and Pt2, DMSO, r.t.; Table S3: CIE 1931 coordinates of Pt1 (in solution, r.t.), and Pt2, Au1−Au3 (solid state) under different conditions; Figure S11: Normalized solid-state emission spectra of Au2 under different conditions; Figure S12: The structures of Pt1−Pt3 complexes optimized by DFT calculations; Figure S13: The structures of Au1–Au3 complexes optimized by DFT calculations; Figure S14: Natural transition orbitals for the active singlet S_3_^*^ of the complex Au2 (294 nm, *f* = 1.77); Figure S15: Natural transition orbitals for the active singlet S_2_^*^ of the complex Au3 (323 nm, *f* = 0.80); Figure S16: Natural transition orbitals for the lowest singlet S_1_ of the complex Au1; Figure S17: Natural transition orbitals for the lowest singlet S_1_ of the complex Au2; Figure S18: Natural transition orbitals for the lowest singlet S_1_ of the complex Au3; Figure S19: Natural transition orbitals for the lowest triplet T_1_ of the complex Au3 (539 nm); Figure S20: Natural transition orbitals for the active singlets S_1_^*^ (449 nm, *f* = 0.1123) and S_2_^*^ (409 nm, *f* = 1.3656) of the complex Pt1; Figure S21: Natural transition orbitals for the active singlets S_1_^*^ (473 nm, *f* = 2.0668) and S_3_^*^ (382 nm, *f* = 0.1551) of the complex Pt2; Figure S22: Optimized structures of the ground (left) and the lowest triplet (right) states for Pt1; Figure S23: Optimized structures of the ground (left) and the lowest triplet (right) states for Pt2
