# Supplementary material for: Does Metal Matter: Comparing Photophysical Properties of Bis-Cyclometalated Alkynylphosphonium Au(III) and Pt(II) Complexes
Source: Molecules. 2025 Jun 2;30(11):2434. doi: 10.3390/molecules30112434 (PMC12155794; doi:10.3390/molecules30112434)
Supplement: Supplementary file 1 [file molecules-30-02434-s001.zip › esi_revised-2.pdf]

Supplementary information

Does metal matter: comparing photophysical properties of bis-cyclometalated alkynylphosphonium Au(III) and Pt(II) complexes

Maksim Luginin, Aleksandra Paderina, Anastasia Sizova, Elena Tupikina and Elena Grachova*

Institute of Chemistry, St Petersburg University, Universitetskii pr. 26, 198504 St. Petersburg, Russia

Correspondence: e.grachova@spbu.ru

Content

Scheme S1. The complexes **Pt1**–**Pt3** and **Au1**–**Au3**..4

XRD structure determination..4

Table S1. Crystallographic data for the compound **Pt1**...5

Table S2. Selected bond angles and distances for the compound **Pt1**...............................6

Figure S1. (A) ^{1}H (top) and ^{31}P{H} (bottom) spectra in aromatic range of **Pt1**; (B) ^{1}H^{1}H COSY spectra in aromatic range of **Pt1**. 7

Figure S2. (A) ^{1}H (top) and ^{31}P{H} (bottom) spectra in aromatic range of **Pt2**; (B) ^{1}H^{1}H COSY spectra in aromatic range of **Pt2**. 8

Figure S3. (A) ^{1}H (top), ^{31}P{H} (middle) and ^{19}F (bottom) spectra in aromatic range of **Au1**; (B) ^{1}H^{1}H COSY spectra in aromatic range of **Au1**...9

Figure S4. (A) ^{1}H (top), ^{31}P{H} (middle) and ^{19}F (bottom) spectra in aromatic range of **Au2**; (B) ^{1}H^{1}H COSY spectra in aromatic range of **Au2**...10

Figure S5. (A) ^{1}H (top), ^{31}P{H} (middle) and ^{19}F (bottom) spectra in aromatic range of **Au3**; (B) ^{1}H^{1}H COSY spectra in aromatic range of **Au3**...11

Figure S6. Experimental (left) ESI^{+} MS spectra of **Pt1**–**Pt3** and simulated (right) isotopic patterns of the $[M+H]^{+}$..12

Figure S7. Experimental (left) ESI^{+} MS spectra of **Au1**–**Au3** and simulated (right) isotopic patterns of the $[M]^{+}$. 13

Figure S8. FTIR spectra of **Pt1**–**Pt3** and **Au1**–**Au3** in the region of C≡C vibration, KBr.... 14

Figure S9. (a) Hirshfeld surface 2D fingerprint plot (all interactions), (b) Hirshfeld surface 2D fingerprint plot (Pt–H interaction), (c) d_{norm} Hirshfeld surface (Pt–H contacts only), and (d) shape index, **Pt1**...14

Figure S10. The low-energy part of the UV-vis spectra of **Pt1** and **Pt2**, DMSO, r.t. 15

Table S3. CIE 1931 coordinates of **Pt1** (in solution, r.t.), and **Pt2**, **Au1**−**Au3** (solid state) under different conditions...15

Figure S11. Normalized solid-state emission spectra of **Au2** under different conditions.... 15

Figure S12. The structures of **Pt1**−**Pt3** complexes optimized by DFT calculations........16

Figure S13. The structures of **Au1**–**Au3** complexes optimized by DFT calculations. 16

Figure S14. Natural transition orbitals for the active singlet S_3^* of the complex **Au2** (294 nm, f = 1.77). 17

Figure S15. Natural transition orbitals for the active singlet S_2^* of the complex **Au3** (323 nm, f = 0.80). 17

Figure S16. Natural transition orbitals for the lowest singlet S_1 of the complex **Au1**. 17

Figure S17. Natural transition orbitals for the lowest singlet S_1 of the complex **Au2**. 18

Figure S18. Natural transition orbitals for the lowest singlet S_1 of the complex **Au3**. 18

Figure S19. Natural transition orbitals for the lowest triplet T_1 of the complex **Au3** (539 nm). 18

Figure S20. Natural transition orbitals for the active singlets S_1^* (449 nm, f = 0.1123) and S_2^* (409 nm, f = 1.3656) of the complex **Pt1**. 19

Figure S21. Natural transition orbitals for the active singlets S_1^* (473 nm, f = 2.0668) and S_3^* (382 nm, f = 0.1551) of the complex **Pt2**. 19

Figure S22. Optimized structures of the ground (left) and the lowest triplet (right) states for **Pt1**. 20

Figure S23. Optimized structures of the ground (left) and the lowest triplet (right) states for **Pt2**. 20

References..20

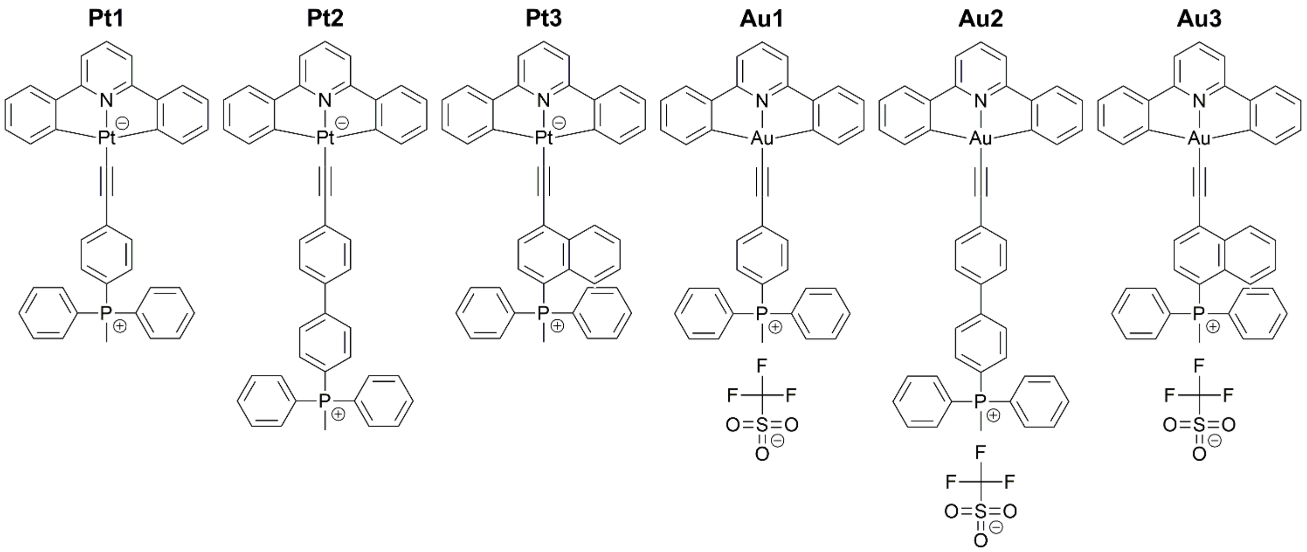

Scheme S1. The complexes **Pt1**–**Pt3** and **Au1**–**Au3**.

XRD structure determination

The crystal structures of **Pt1** was determined by the means of single crystal X-ray diffraction analysis. Crystals were fixed on a micro mounts and the diffraction data have been collected on the Rigaku Oxford Diffraction diffractometer at a temperature of 100K. Crystal of **Pt1** was placed on the Rigaku XtaLAB SuperNova diffractometer and measured using monochromated Cu K_{α} radiation. Data were integrated and corrected for background, Lorentz, and polarization effects. An empirical absorption correction based on spherical harmonics implemented in the SCALE3 ABSPACK algorithm was applied in *CrysAlisPro* program.[1] The unit-cell parameters (Table S1) were refined by the least-squares techniques. The structure were solved by dual-space algorithm and refined using the *SHELX* programs[2,3] incorporated in the *OLEX2* program package.[4,5] The crystal of **Pt1** was found to be non-merohedrally twinned (K = Mean(F_o^2)/Mean(F_c^2)>13; for all the "most disagreeable reflections" F_o is much greater than F_c; high R-values after the refinement although R_{int} is ca. 4%). The structure was refined using the HKLF5 routine, with the twin law (1.000 0 0.976, 0 −1 0, 0 0 −1) and BASF parameter of 0.199(2). The final models included coordinates and anisotropic displacement parameters for all non-H atoms. The carbon-bound H atoms were placed in calculated positions and were included in the refinement in the 'riding' model approximation, U_{iso}(H) set to 1.5U_{eq}(C) and C–H 0.96 Å for the CH_3 groups and U_{iso}(H) set to 1.2U_{eq}(C) and C–H 0.93 Å for the CH groups. Supplementary crystallographic data for this paper have been deposited at Cambridge Crystallographic Data Centre (CCDC 2383597) and can be obtained free of charge via www.ccdc.cam.ac.uk/structures/.

Table S1. Crystallographic data for the compound **Pt1**.

**Compound**	**Pt1**
Formula	$C_{79}H_{62}N_2OP_2Pt_2$
Crystal system	monoclinic
a (Å)	24.9558(8)
b (Å)	12.2843(3)
c (Å)	21.9034(7)
α (°)	90
β (°)	115.373(4)
γ (°)	90
V (Å^3)	6067.1(4)
Molecular weight	1507.42
Space group (number)	$I2/c$ (15)
μ (mm^{-1})	9.385
Temperature (K)	100.00(10)
Z	4
ρ_{calc} (g/cm^{-3})	1.650
Crystal size (mm^3)	0.08×0.14×0.18
Diffractometer	SuperNova, Single source at offset/far, HyPix3000
Radiation	Cu K_α
Total reflections	5420
Unique reflections	5420
Angle range 2Θ (°)	7.84 to 138.18 (0.83 Å)
Reflections with $\|F_o\| \geq 4\sigma_F$	5196
R_{int}	0.0395
R_σ	0.0348
R_1 ($\|F_o\| \geq 4\sigma_F$)	0.0482
wR_2 ($\|F_o\| \geq 4\sigma_F$)	0.1425
R_1 (all data)	0.0494
wR_2 (all data)	0.1441
Goodness-of-fit on F^2	1.074
ρ_{max}, ρ_{min} (e/Å^3)	3.83/−1.41
CCDC number	2383597

Table S2. Selected bond angles and distances for the compound **Pt1**.

**Bond lengths, Å**		**Bond angles, °**	
Pt1–N1	2.016(6)	C1–Pt1–C17	160.5(3)
Pt1–C1	2.064(7)	N1–Pt1–C1	80.4(3)
Pt1–C17	2.063(7)	N1–Pt1–C17	80.1(3)
Pt1–C18	1.944(7)	C1–Pt1–C18	99.1(3)
C18–C19	1.228(10)	C17–Pt1–C18	100.3(3)

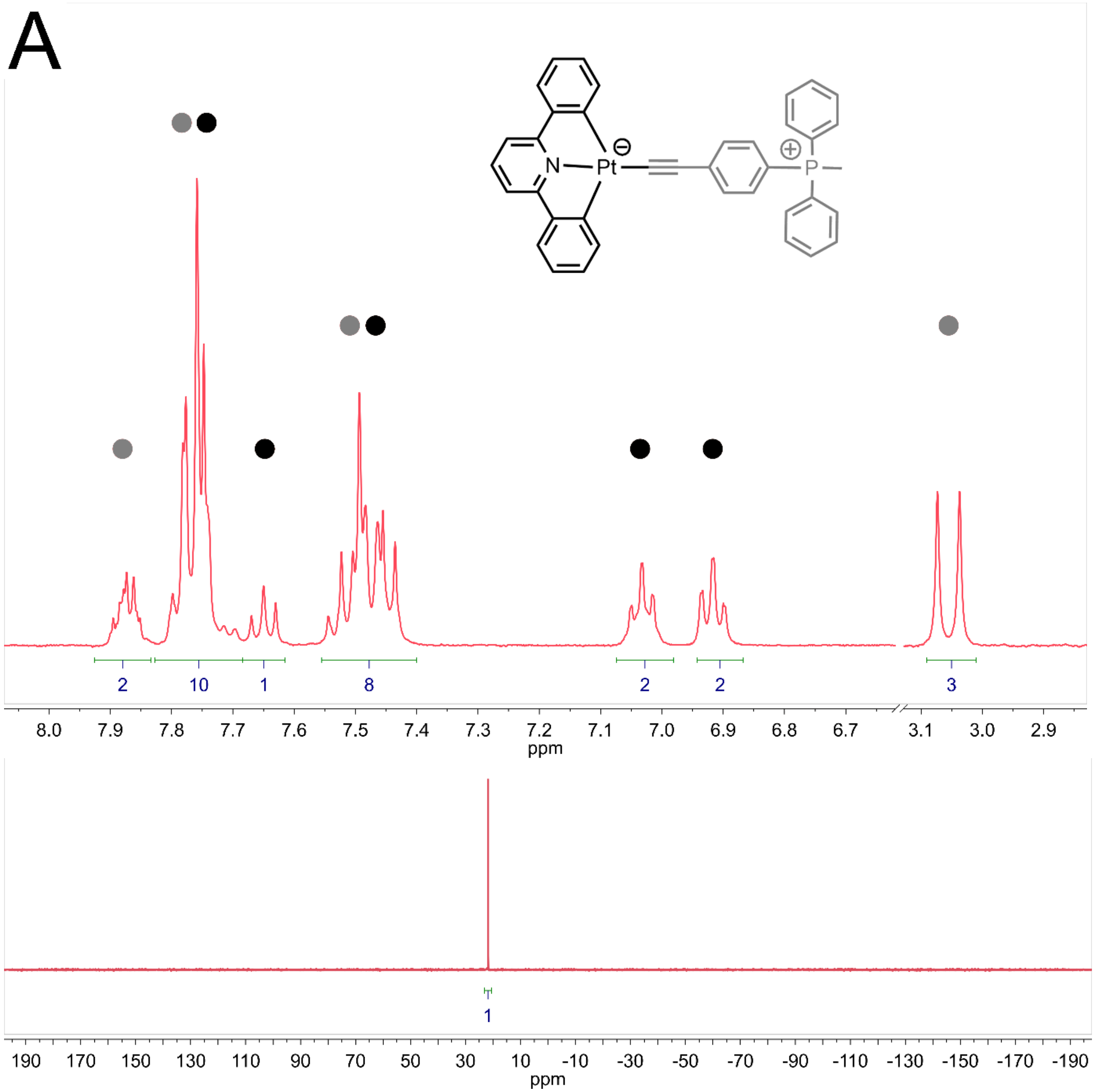

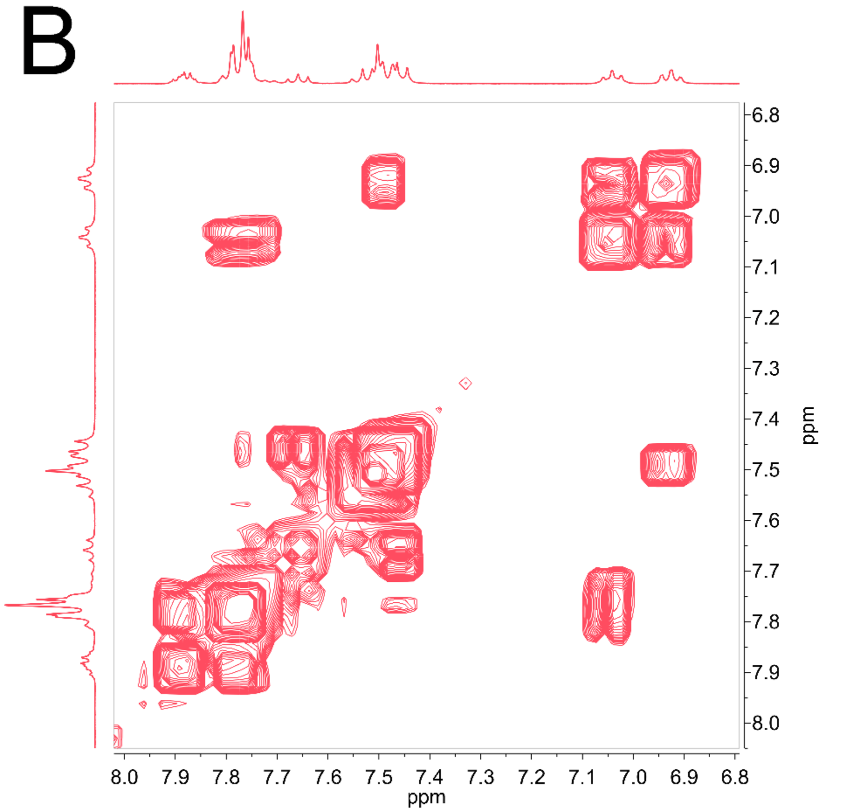

Figure S1. (A) ^{1}H (top) and $^{31}P\{H\}$ (bottom) spectra in aromatic range of **Pt1**; (B) $^{1}H^{1}H$ COSY spectra in aromatic range of **Pt1**. All spectra were measured at 298K in DMSO-d_6.

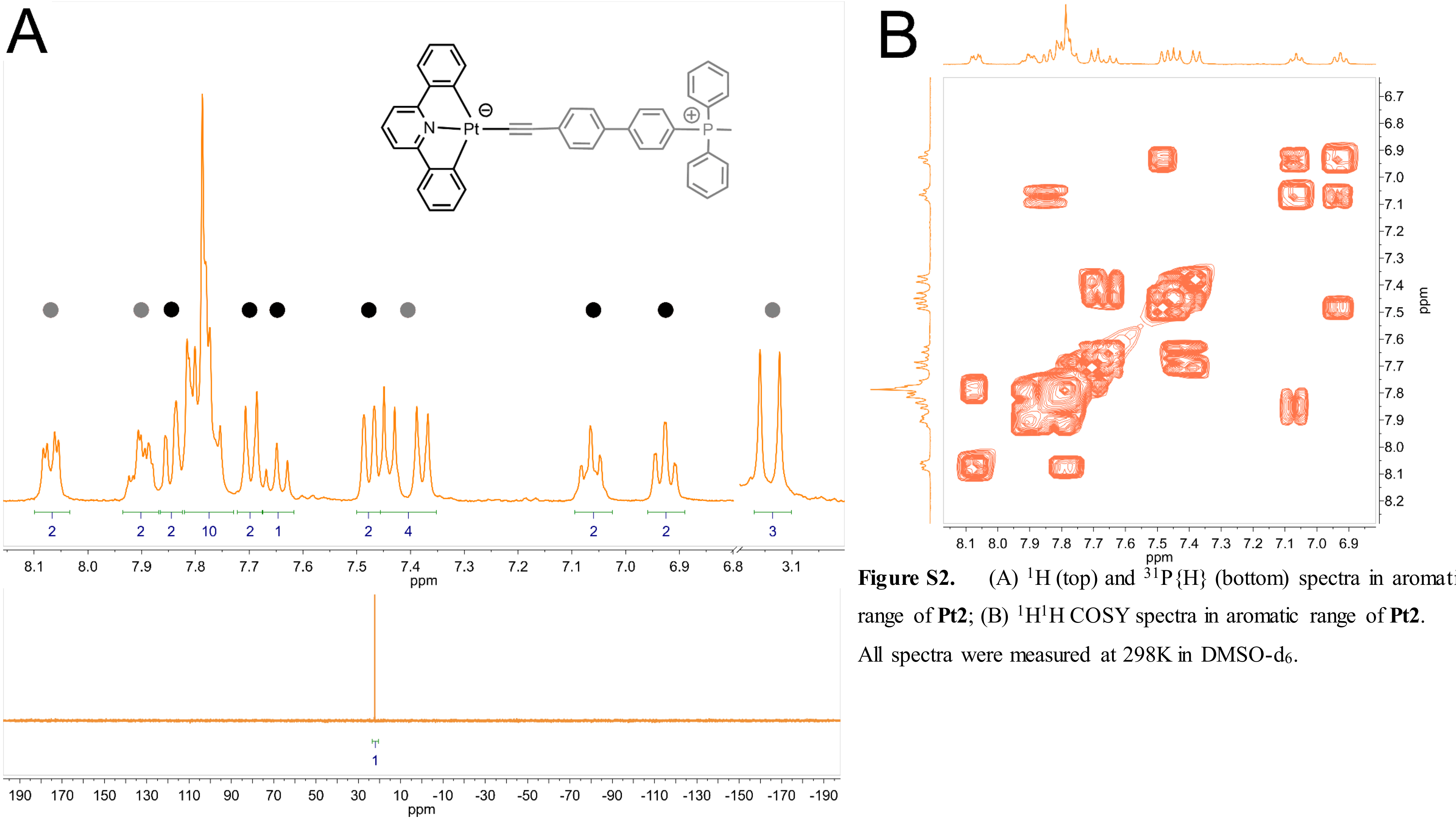

Figure S2. (A) ^{1}H (top) and ^{31}P{H} (bottom) spectra in aromatic range of **Pt2**; (B) ^{1}H^{1}H COSY spectra in aromatic range of **Pt2**. All spectra were measured at 298K in DMSO-d_6.

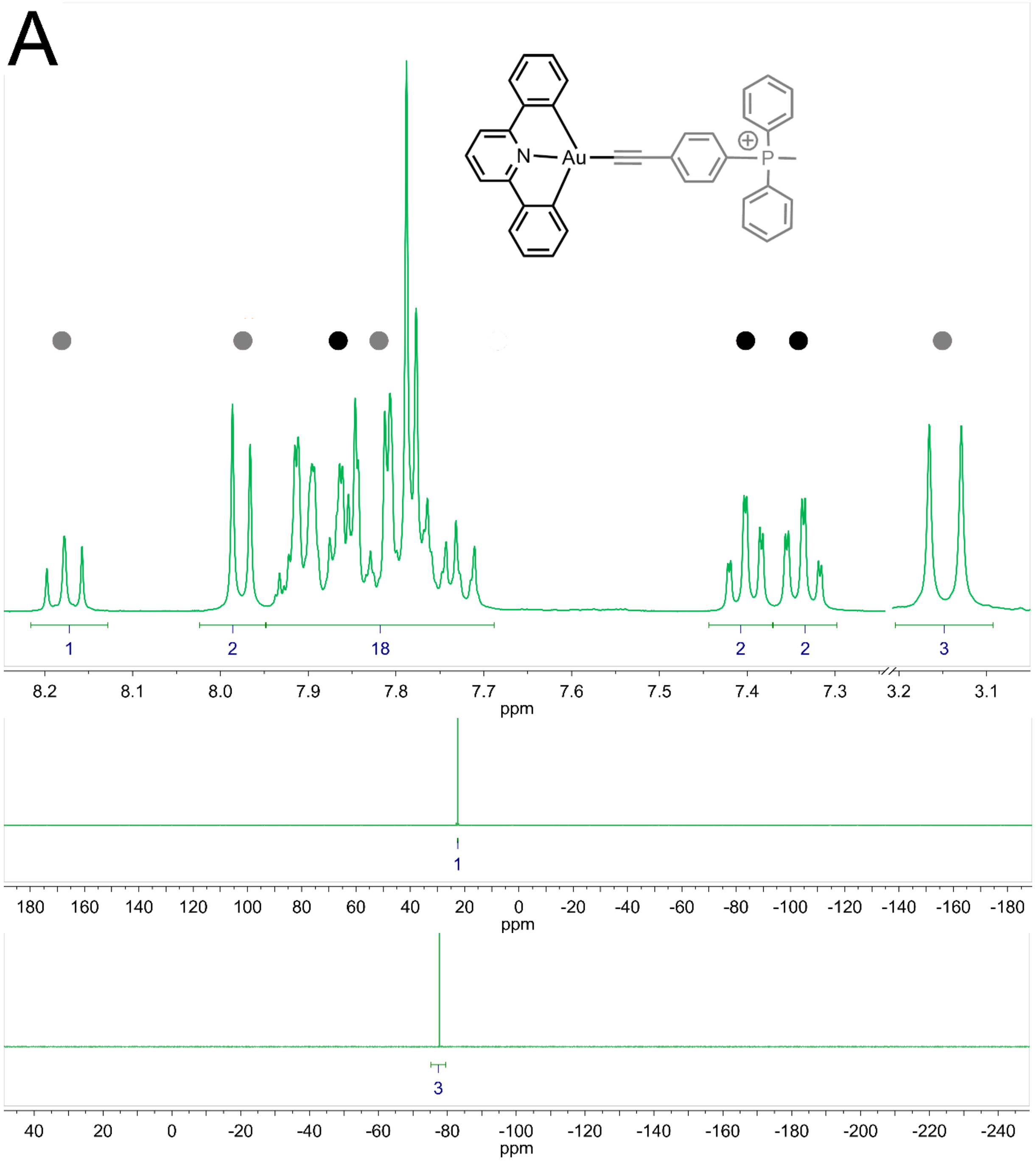

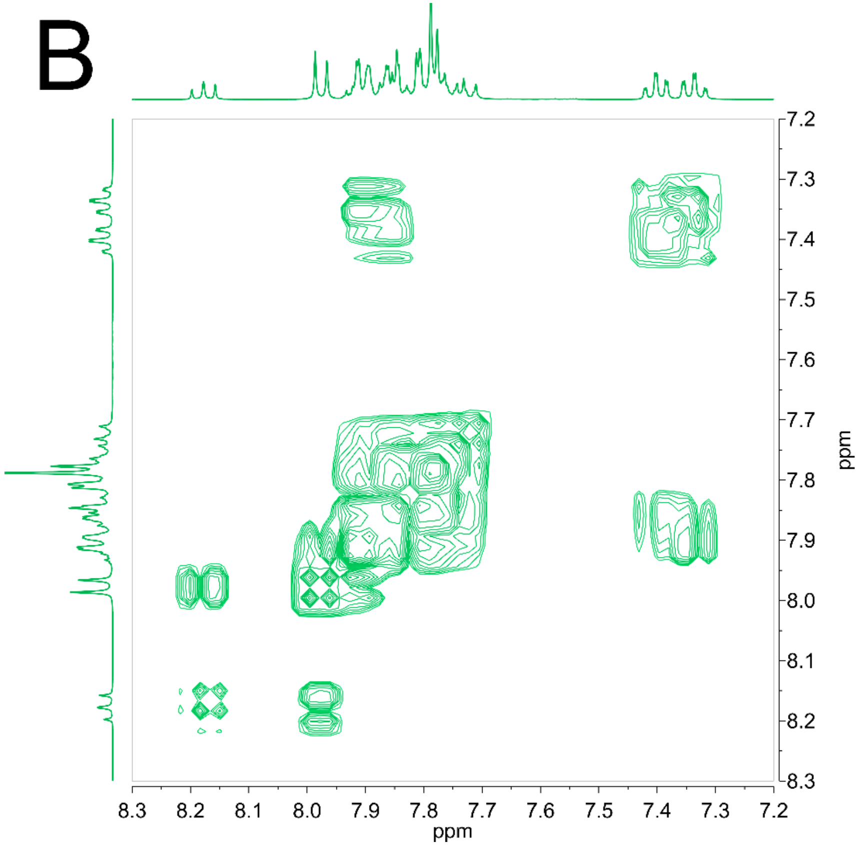

Figure S3. (A) ^{1}H (top), $^{31}P\{H\}$ (middle) and ^{19}F (bottom) spectra in aromatic range of **Au1**; (B) $^{1}H^{1}H$ COSY spectra in aromatic range of **Au1**.

All spectra were measured at 298K in DMSO-d_6.

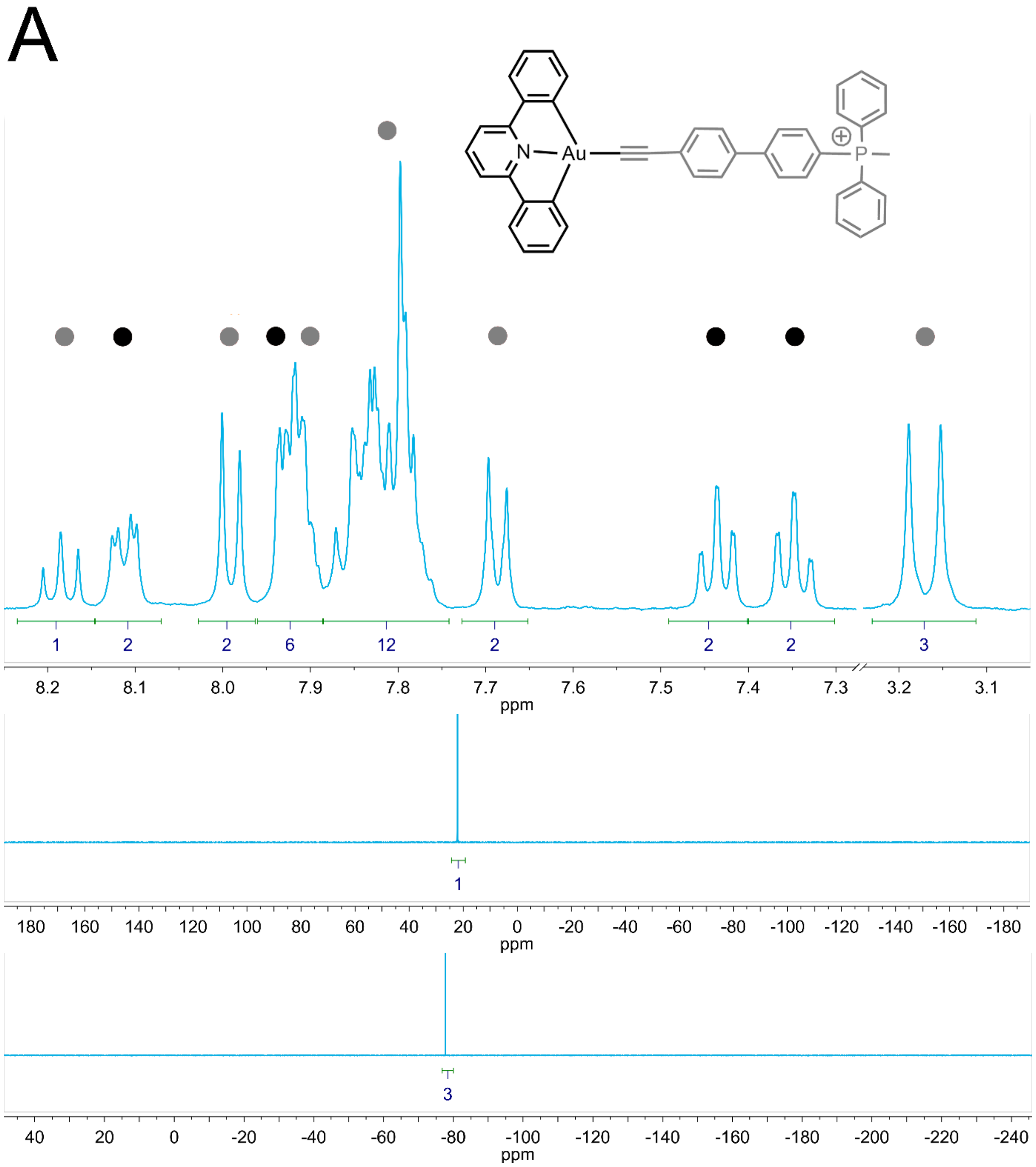

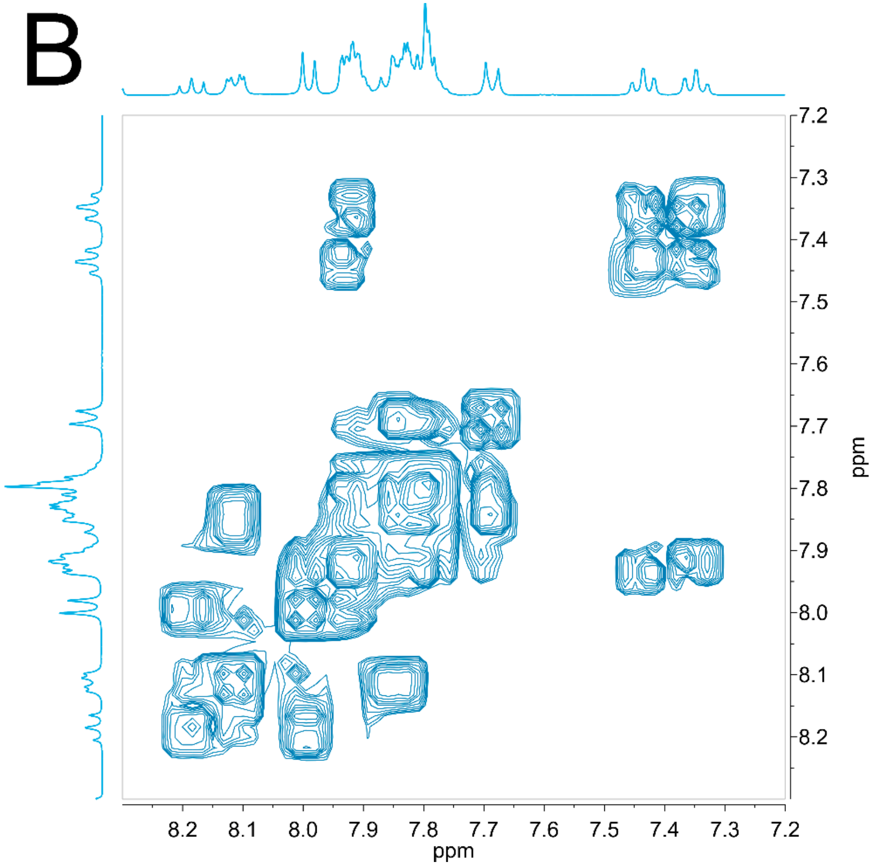

Figure S4. (A) ^{1}H (top), $^{31}P\{H\}$ (middle) and ^{19}F (bottom) spectra in aromatic range of **Au2**; (B) $^{1}H^{1}H$ COSY spectra in aromatic range of **Au2**.

All spectra were measured at 298K in DMSO-d_6.

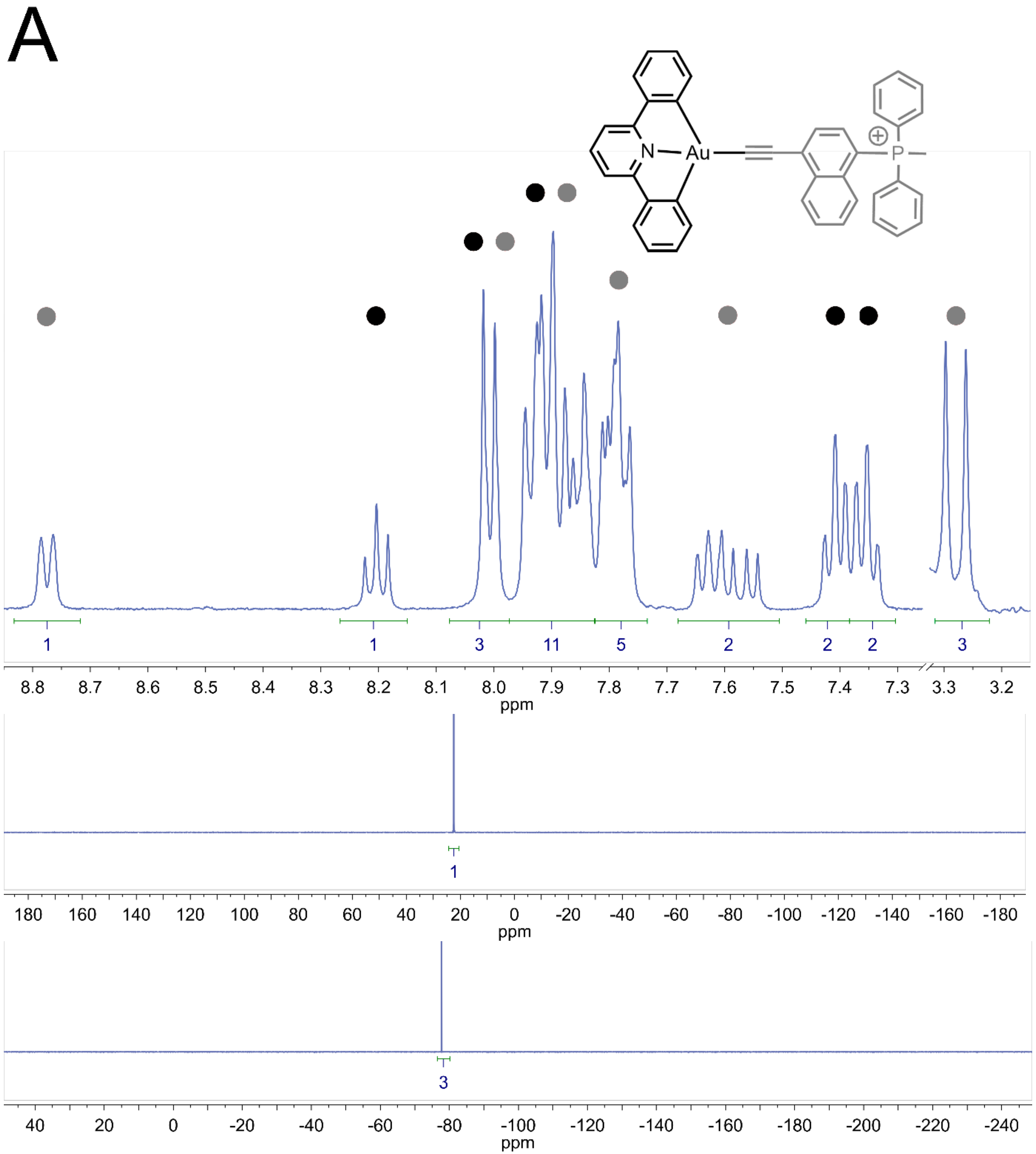

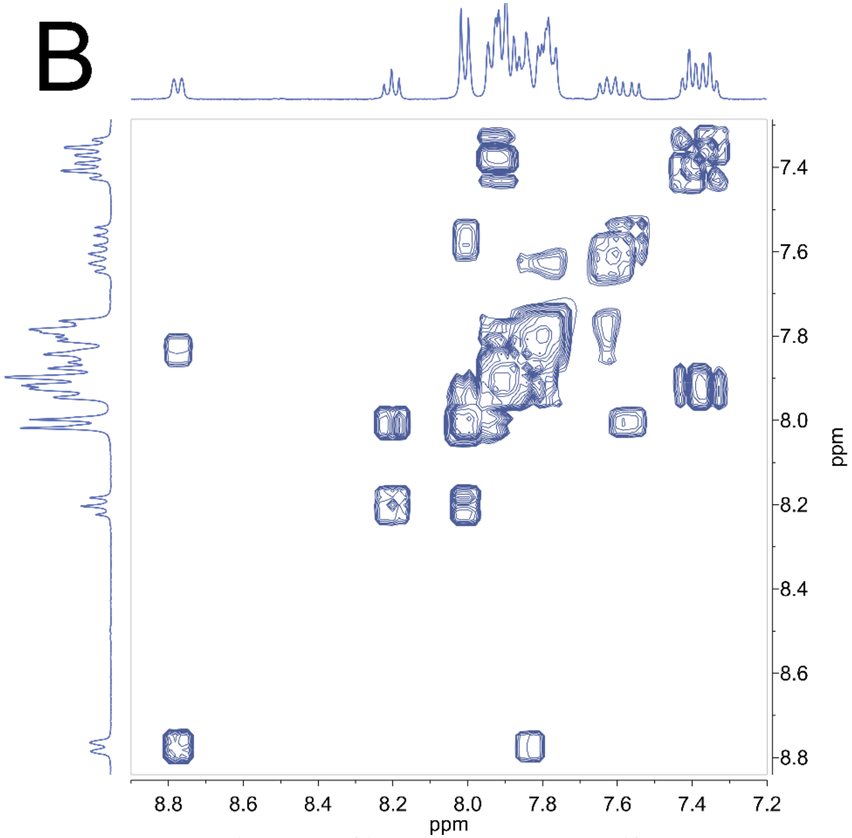

Figure S5. (A) ^{1}H (top), ^{31}P{H} (middle) and ^{19}F (bottom) spectra in aromatic range of **Au3**; (B) ^{1}H^{1}H COSY spectra in aromatic range of **Au3**.

All spectra were measured at 298K in DMSO-d_6.

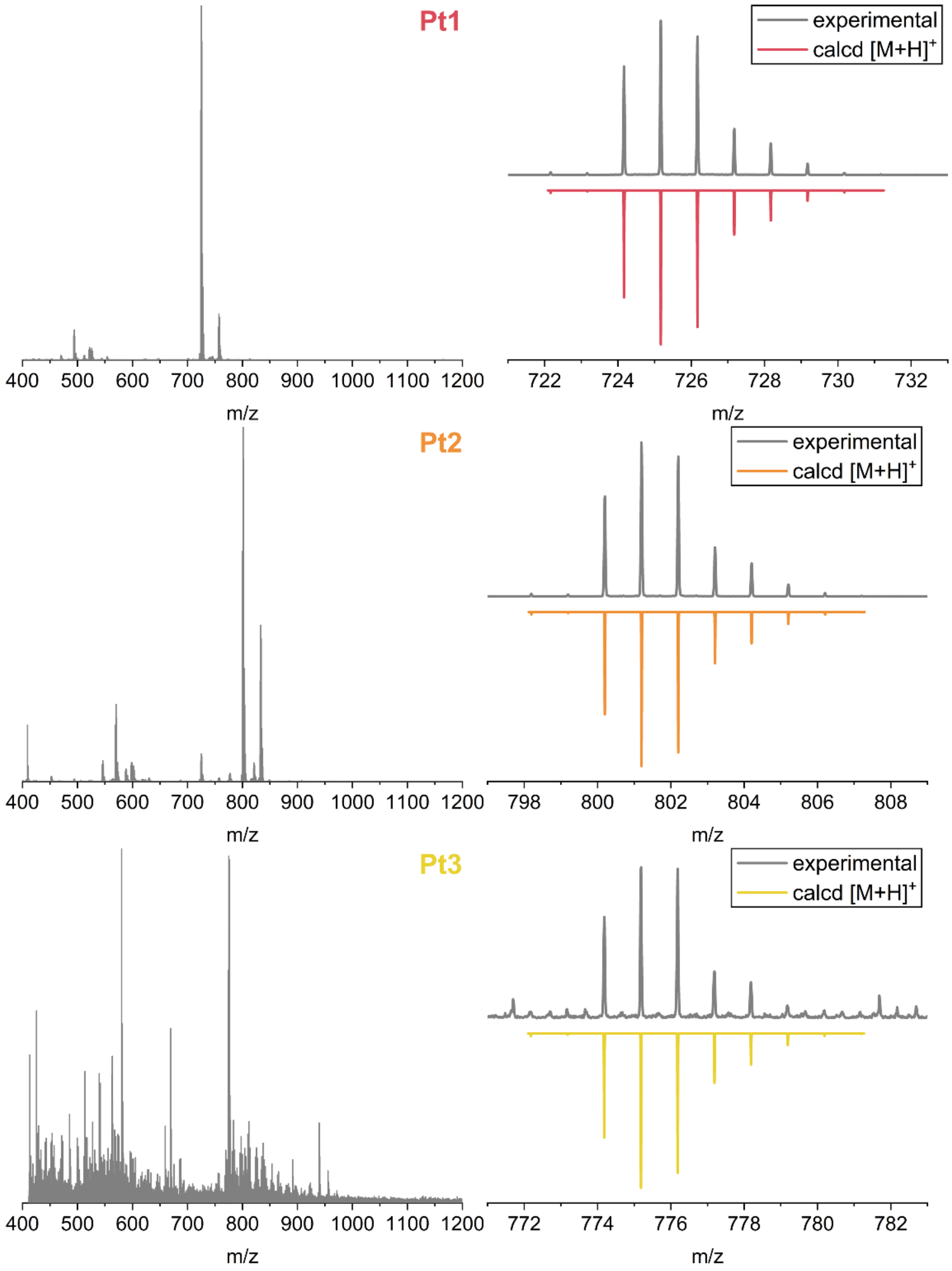

Figure S6. Experimental (left) ESI$^+$ MS spectra of **Pt1**–**Pt3** and simulated (right) isotopic patterns of the $[M+H]^+$.

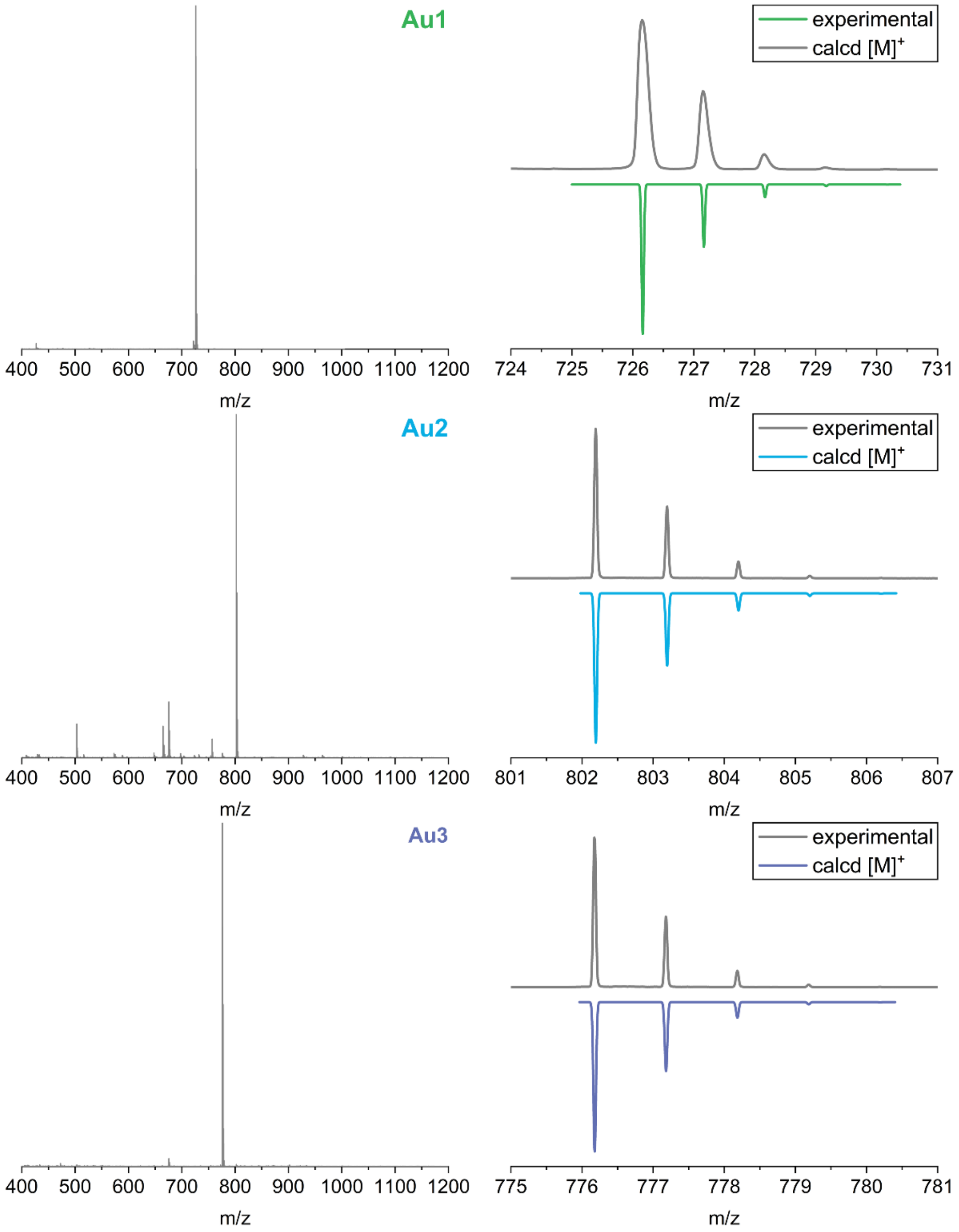

Figure S7. Experimental (left) ESI$^+$ MS spectra of **Au1**–**Au3** and simulated (right) isotopic patterns of the $[M]^+$.

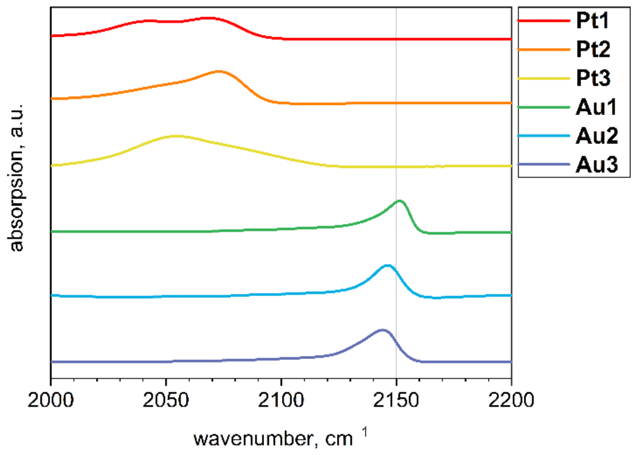

Figure S8. FTIR spectra of **Pt1**–**Pt3** and **Au1**–**Au3** in the region of C≡C vibration, KBr.

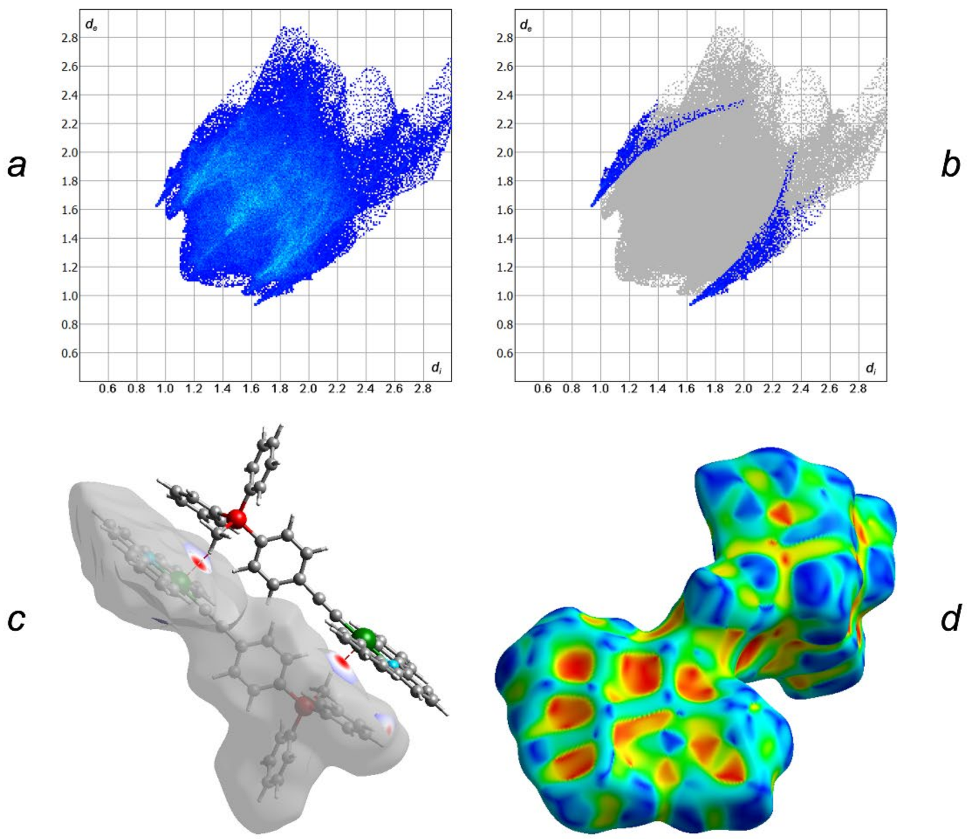

Figure S9. (a) Hirshfeld surface 2D fingerprint plot (all interactions), (b) Hirshfeld surface 2D fingerprint plot (Pt–H interaction), (c) d_{norm} Hirshfeld surface (Pt–H contacts only), and (d) shape index, **Pt1**.

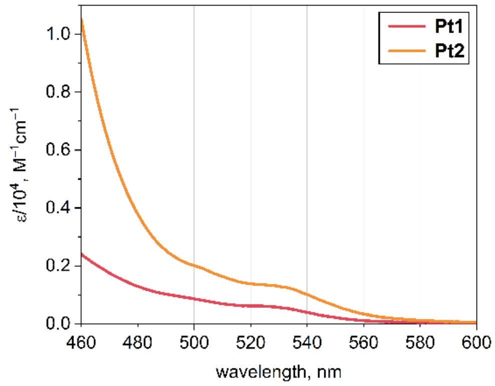

Figure S10. The low-energy part of the UV-vis spectra of **Pt1** and **Pt2**, DMSO, r.t.

Table S3. CIE 1931 coordinates of **Pt1** (in solution, r.t.), and **Pt2**, **Au1**−**Au3** (solid state) under different conditions.

Compound	Conditions	X	Y
Au1	solid state state, r.t.	0.47	0.51
Au1	solid state, 77 K	0.41	0.55
Au2	solid state, r.t.	0.46	0.52
Au2	solid state, 77 K	0.37	0.58
Au3	solid state, r.t.	0.48	0.51
Au3	solid state, 77 K	0.53	0.47
Pt1	DMSO solution, r.t.	0.29	0.59
Pt1	solid state, r.t.	0.59	0.41
Pt1	solid state, 77 K	0.67	0.33
Pt2	solid state, r.t.	0.61	0.39
Pt2	solid state, 77 K	0.65	0.35

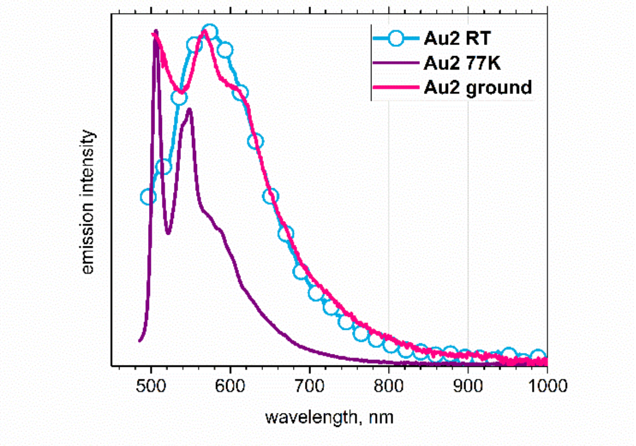

Figure S11. Normalized solid-state emission spectra of **Au2** under different conditions.

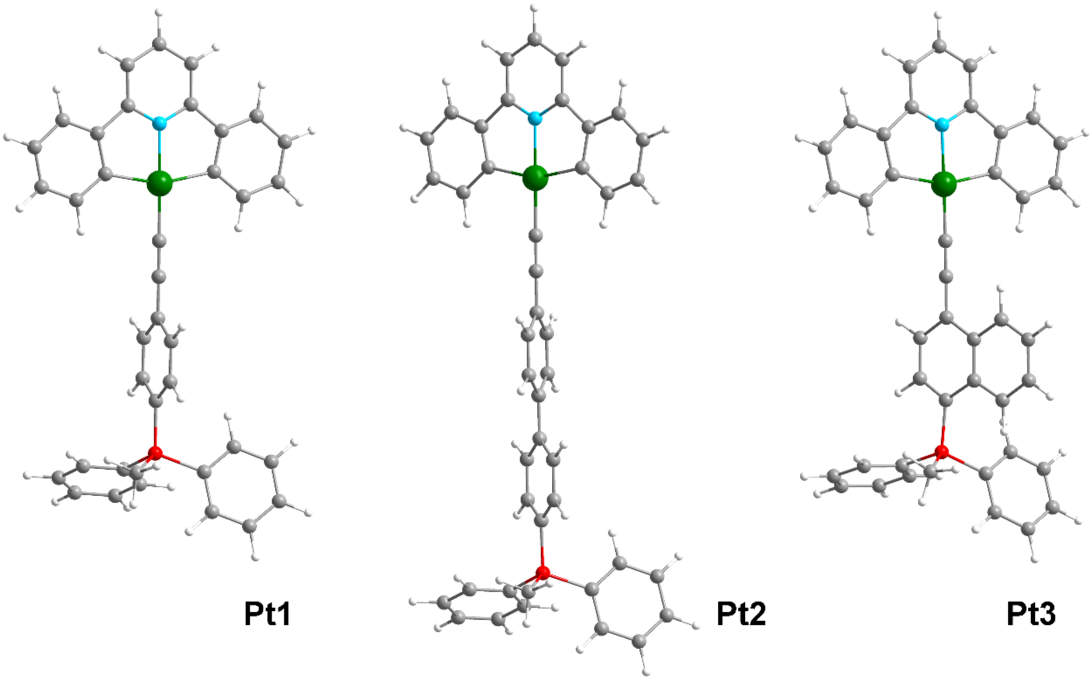

Figure S12. The structures of **Pt1−Pt3** complexes optimized by DFT calculations.

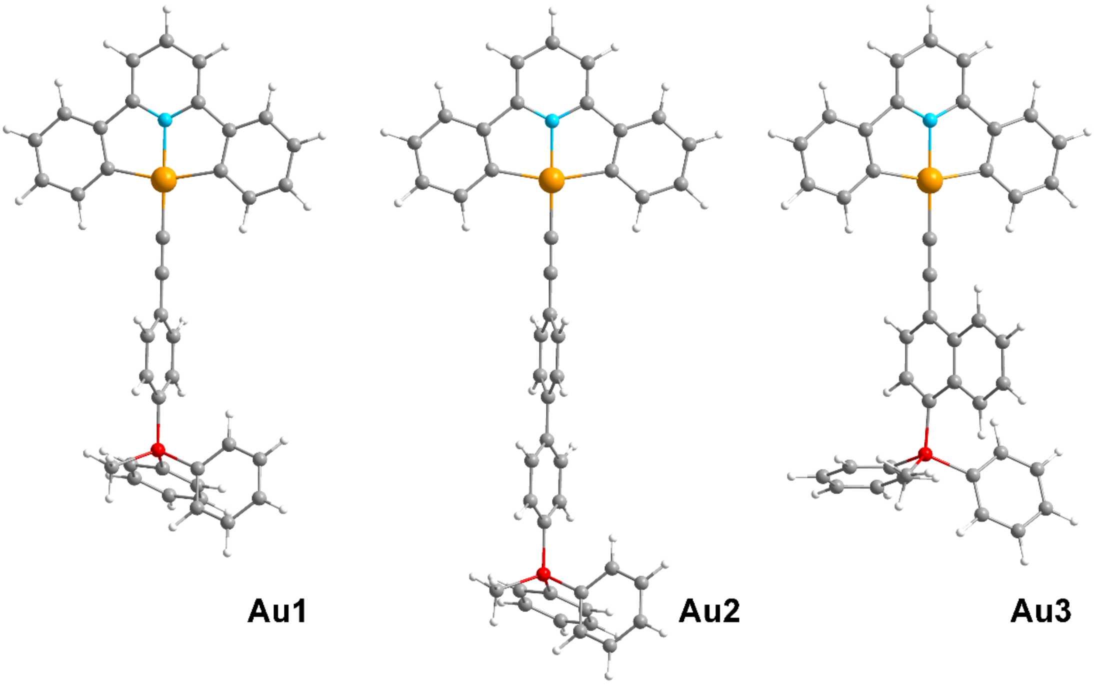

Figure S13. The structures of **Au1–Au3** complexes optimized by DFT calculations.

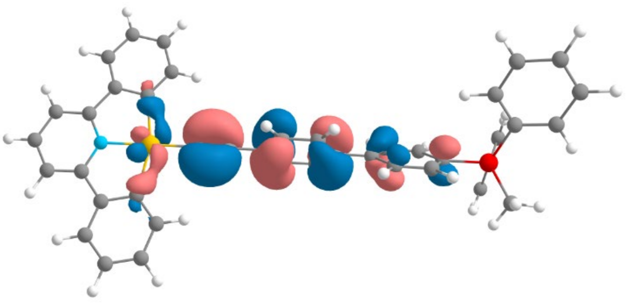

Figure S14. Natural transition orbitals for the active singlet S_3^* of the complex **Au2** (294 nm, f = 1.77).

NTO **NTO***

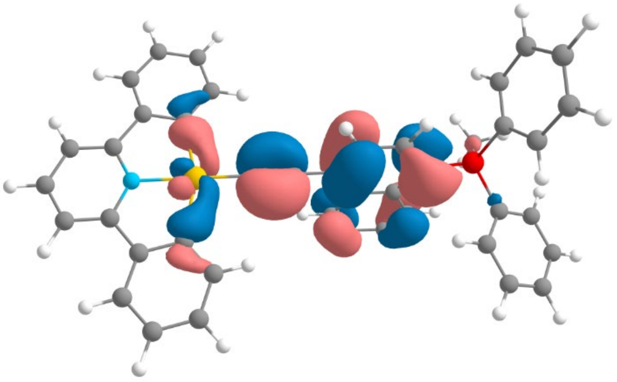

Figure S15. Natural transition orbitals for the active singlet S_2^* of the complex **Au3** (323 nm, f = 0.80).

NTO **NTO***

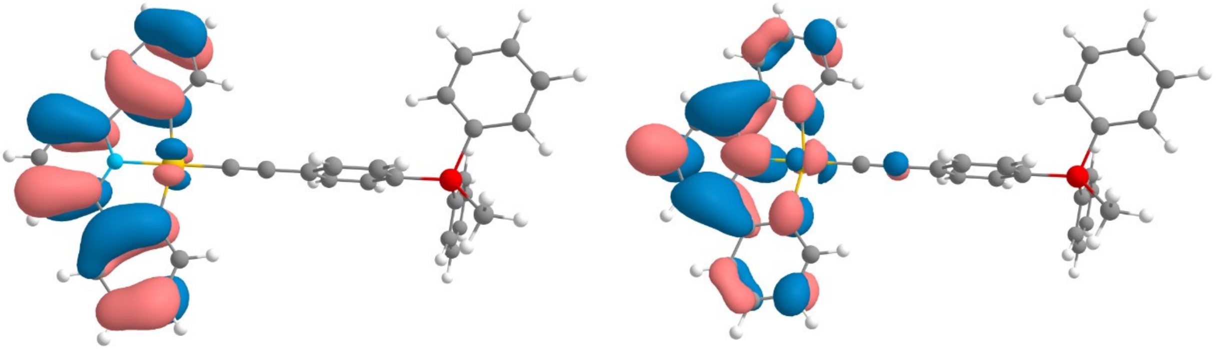

Figure S16. Natural transition orbitals for the lowest singlet S_1 of the complex **Au1**.

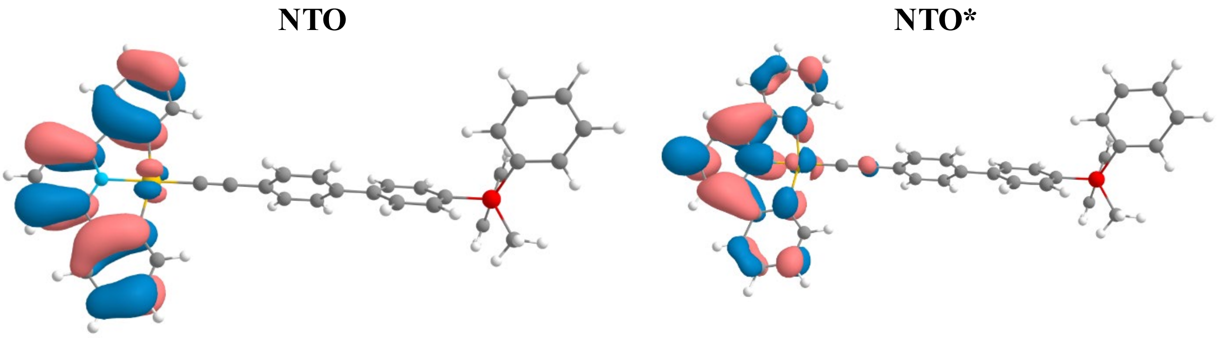

Figure S17. Natural transition orbitals for the lowest singlet S_1 of the complex **Au2**.

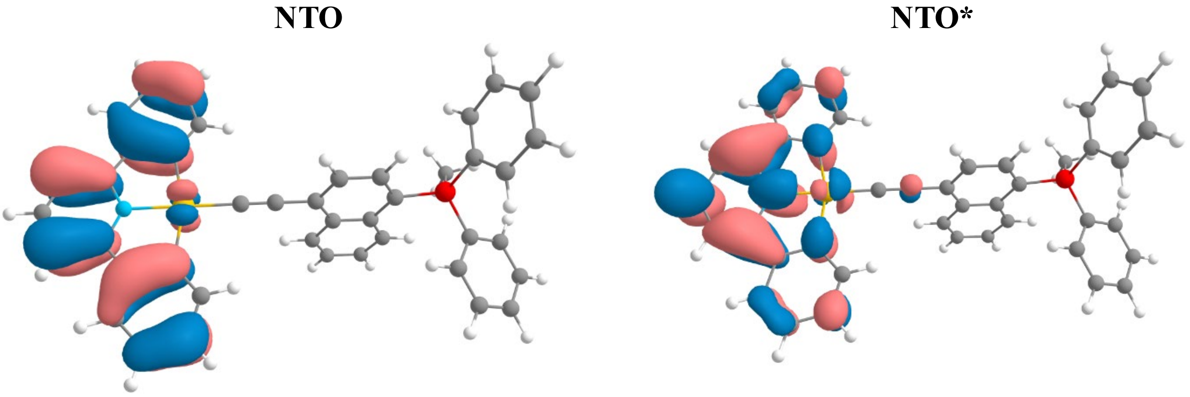

Figure S18. Natural transition orbitals for the lowest singlet S_1 of the complex **Au3**.

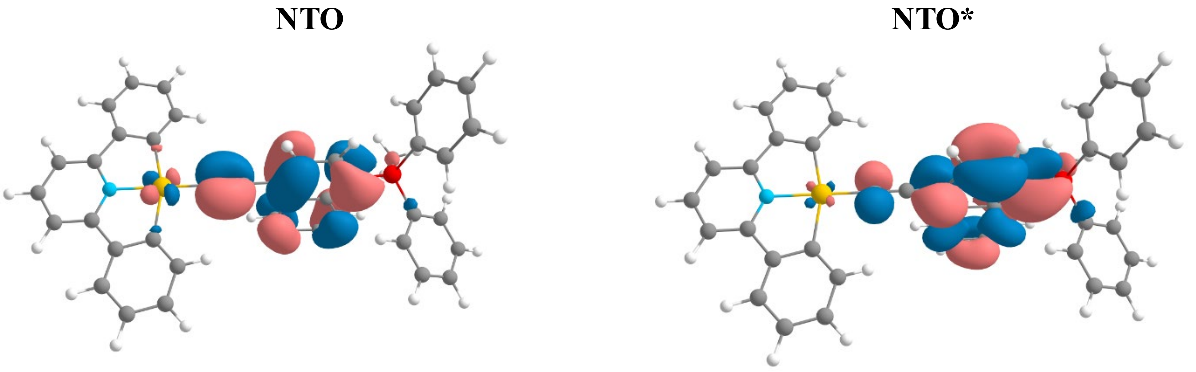

Figure S19. Natural transition orbitals for the lowest triplet T_1 of the complex **Au3** (539 nm).

NTO NTO*

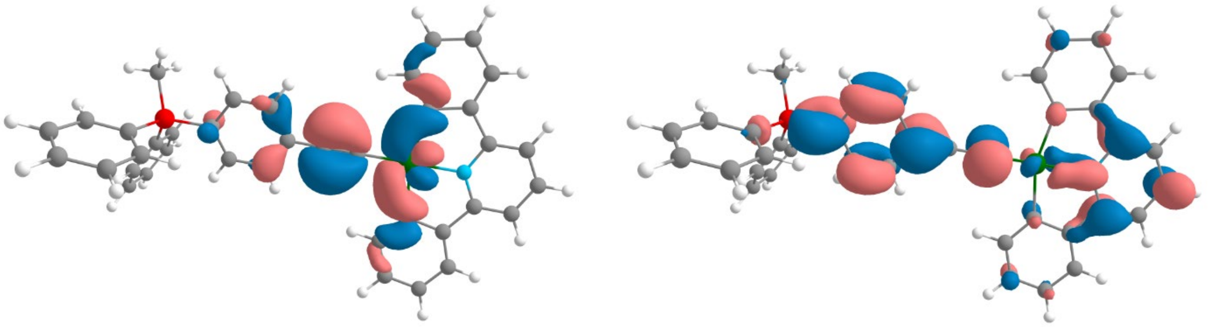

S_1*

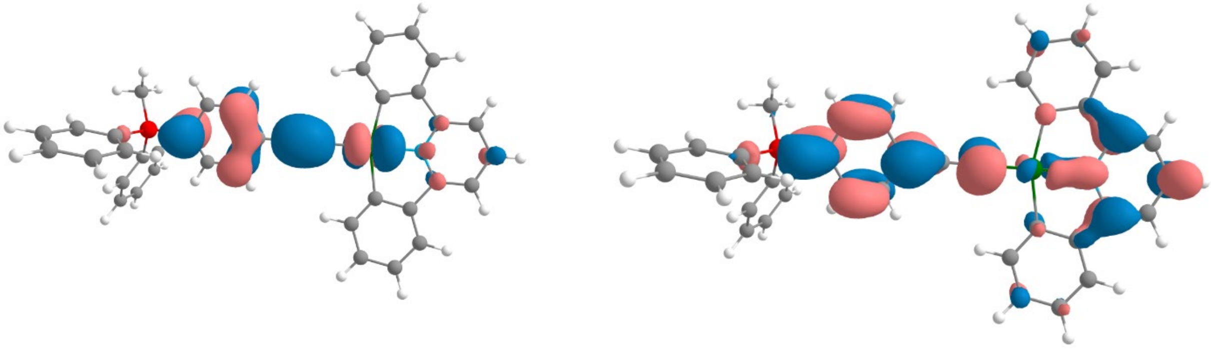

S_2*

Figure S20. Natural transition orbitals for the active singlets S_1^* (449 nm, f = 0.1123) and S_2^* (409 nm, f = 1.3656) of the complex **Pt1**.

NTO NTO*

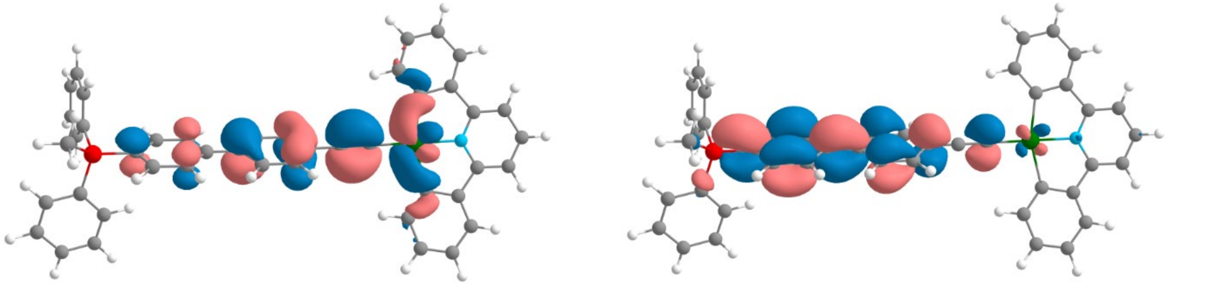

S_1*

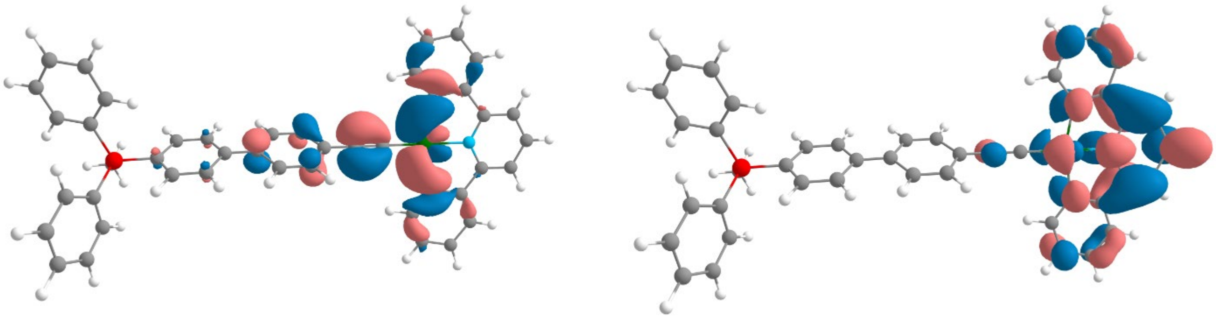

S_3*

Figure S21. Natural transition orbitals for the active singlets S_1^* (473 nm, f = 2.0668) and S_3^* (382 nm, f = 0.1551) of the complex **Pt2**.

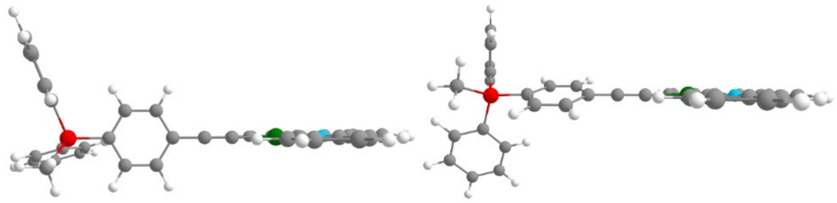

Figure S22. Optimized structures of the ground (left) and the lowest triplet (right) states for **Pt1**.

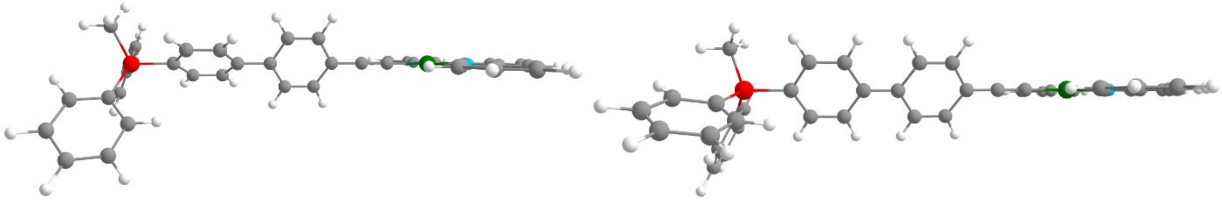

Figure S23. Optimized structures of the ground (left) and the lowest triplet (right) states for **Pt2**.

References

1. CrysAlisPro, Rigaku Oxford Diffraction. *CrysAlisPro, Rigaku Oxford Diffr.* 2017, Version 1.171.39.35a, 2017.
2. Sheldrick, G.M. SHELXT - Integrated space-group and crystal-structure determination. *Acta Crystallogr. Sect. A Found. Crystallogr.* **2015**, *71*, 3–8, doi:10.1107/S2053273314026370.
3. Sheldrick, G.M. Crystal structure refinement with SHELXL. *Acta Crystallogr. Sect. C* **2015**, *71*, 3–8, doi:10.1107/S2053229614024218.
4. Bourhis, L.J.; Dolomanov, O. V.; Gildea, R.J.; Howard, J.A.K.; Puschmann, H. The anatomy of a comprehensive constrained, restrained refinement program for the modern computing environment – Olex2 dissected. *Acta Crystallogr. Sect. A Found. Adv.* **2015**, *71*, 59–75, doi:10.1107/S2053273314022207.
5. Dolomanov, O. V.; Bourhis, L.J.; Gildea, R.J.; Howard, J.A.K.; Puschmann, H. OLEX2 : a complete structure solution, refinement and analysis program. *J. Appl. Crystallogr.* **2009**, *42*, 339–341, doi:10.1107/S0021889808042726.
